# Shared Plasma Metabolites Mediate Causal Effects of Metabolic Diseases on Colorectal Cancer: A Two-Step Mendelian Randomization Study

**DOI:** 10.3390/biomedicines13102433

**Published:** 2025-10-06

**Authors:** Xinyi Shi, Yuxin Tang, Yu Zhang, Yu Cheng, Yingying Ma, Fangrong Yan, Tiantian Liu

**Affiliations:** School of Science, China Pharmaceutical University, Nanjing 210009, China; 3323051545@stu.cpu.edu.cn (X.S.); 3223051531@stu.cpu.edu.cn (Y.T.); 3223051474@stu.cpu.edu.cn (Y.Z.); 3122054212@stu.cpu.edu.cn (Y.C.); 3323051550@stu.cpu.edu.cn (Y.M.)

**Keywords:** colorectal cancer, plasma metabolites, metabolic diseases, mendelian randomisation, fatty-acid metabolism

## Abstract

**Background**: Colorectal cancer (CRC) is significantly associated with multiple metabolic diseases, with plasma metabolites potentially mediating this relationship. This large-scale metabolomics study aims to (1) quantify the genetic correlations and causal effects between 10 metabolic disease-related phenotypes and CRC risk; (2) identify the plasma metabolites mediating these effects; and (3) explore downstream regulatory genes and druggable targets. **Methods**: Using linkage disequilibrium score regression and two-sample Mendelian randomization, we assessed the causal relationships between each metabolic trait and CRC. A total of 1091 plasma metabolites and 309 metabolite ratios were identified and analyzed for mediating effects by a two-step MR approach. Colocalization analyses evaluated shared genetic loci. The findings were validated in the UK Biobank for metabolite-trait associations. The expression of candidate genes was explored using data from TCGA, GTEx, and GEO. A *FADS1*-centered protein–protein interaction (PPI) network was constructed via STRING. **Results**: BMI, waist circumference, basal metabolic rate, insulin resistance and metabolic syndrome exhibited both genetic correlation and causal effects on CRC. Five plasma metabolites—mannonate, the glucose/mannose ratio, plasma free asparagine, 1-linolenoyl-2-linolenoyl-GPC (18:2/18:3), and the mannose/trans-4-hydroxyproline ratio—were identified as shared central mediators. A colocalization analysis showed *rs174546* linked CRC and 1-linolenoyl-2-linoleoyl-GPC. Validation in the UK Biobank confirmed the associations between phosphatidylcholine (the lipid class of this metabolite), adiposity measures, and CRC risk. An integrative analysis of TCGA, GTEx, and GEO revealed consistent upregulation of *FADS1/2/3* and *FEN1* in CRC, with high *FADS1* expression predicting a poorer prognosis and showing the distinct cell-type expression in adipose and colon tissue. The PPI network mapping uncovered nine *FADS1* interacting proteins targeted by supplements such as *α*-linolenic acid and eicosapentaenoic acid. **Conclusions**: This study systematically reveals, for the first time, the shared intermediary plasma metabolites and their regulatory genes in the causal pathway from metabolic diseases to CRC. These findings provide candidate targets for subsequent functional validation and biomarker development.

## 1. Introduction

Colorectal cancer (CRC) is a malignant tumor of the gastrointestinal tract that is predominantly adenocarcinomatous, with more than 90% of cases originating from adenoepithelial cells of the colon and rectum [1]. According to global data from the International Agency for Research on Cancer (IARC) of the World Health organization, CRC had approximately 1.9 million new cases and 900,000 deaths in 2020, making it the third most common cancer after lung and breast cancer and the second leading cause of cancer-related mortality worldwide. The number of new cases is projected to rise to 3.2 million globally by 2040 [2]. Studies have shown that about 40–50% of CRC cases are attributable to modifiable environmental factors (e.g., smoking, alcohol consumption, obesity, and poor dietary habits), while genetic factors account for about 30%. Therefore, in-depth analyses of these environmental and genetic factors are important for early diagnosis and improved prognosis [1]. Although traditional risk factors, such as inflammatory bowel disease and high red meat diets, have been extensively studied, they still fail to fully explain the disease mechanisms. In recent years, due to the prevalence of metabolic diseases such as metabolic syndrome (MetS), insulin resistance (IR), and lipid metabolism dysregulation, more and more studies have begun to focus on the potential role of metabolic diseases in CRC [3,4,5,6,7]. Recent observational studies have shown that obese patients have a 109% increased risk of CRC compared with normal-weight populations, and previous observational studies may have underestimated the true strength of the association between body mass index (BMI) and CRC due to the confounding of pre-diagnostic weight changes [8]. In addition, a large prospective cohort study showed that the incidence of CRC was also significantly higher in patients with type 2 diabetes mellitus (T2DM) [9]. Mechanistic studies have shown that T2DM contributes to CRC development through a complex oncogenic network involving metabolic reprogramming, hormonal imbalance, oxidative stress, and epigenetic alterations, which collectively promote CRC cell proliferation, invasion, and immune evasion [10,11]. A large meta-analysis indicated that patients with non-alcoholic fatty liver disease (NAFLD) have a 1.5–2-fold increased risk of gastrointestinal cancers compared to healthy populations [12]. Further metabolomics studies have shown that patients with metabolic diseases have characteristic changes in plasma metabolite profiles, which may play an important role in the development of CRC [13]. Specifically, these metabolites may influence CRC development through mechanisms such as regulating the malignant transformation of intestinal epithelial cells or mediating systemic inflammation, suggesting that plasma metabolites may be key mediators linking metabolic diseases to CRC. However, current studies face important limitations. First, most of the available evidence is derived from observational studies, which are inherently limited in their ability to infer causality and are prone to confounding by factors such as dietary patterns and genetic background. Second, the plasma metabolite profiles shared between metabolic diseases and CRC and their intermediary roles in causal pathways have not yet been systematically elucidated. To address the above issues, there is an urgent need for research strategies with stronger causal inference capabilities to elucidate the role of plasma metabolites in CRC pathogenesis.

Mendelian randomization (MR) is a widely used causal inference approach that leverages genetic variation. Its core principle is to use genetic variants strongly associated with specific exposure factors as instrumental variables (IVs), thereby mimicking the random allocation of exposures in randomized controlled trials (RCTs) [14]. As genetic variants are randomly assigned during meiosis and gamete formation in keeping with the “Mendelian laws of inheritance”, their associations with exposure factors are independent of traditional confounders (e.g., lifestyle, environmental exposures) and are not subject to reverse causation of the study results. This allows MR to effectively mitigate the confounding bias and measurement errors that often limit observational studies [15].

Accordingly, the present study aims to investigate the causal pathways between metabolic diseases and CRC using MR, with a particular focus on the potential mediating role of plasma metabolites. We propose the hypothesis of “shared plasma metabolite mediators”, which suggests that different metabolic diseases may regulate a common set of plasma metabolite profiles to form a unified oncogenic pathway, which ultimately affects the risk of CRC. To validate this hypothesis, we conducted a two-step, two-sample Mendelian randomization (TSMR) analysis using CRC genome-wide association study (GWAS) data obtained from the IEU OpenGWAS (MRC Integrative Epidemiology Unit Open GWAS project) and GWAS Catalog (The NHGRI-EBI GWAS Catalog) databases. This approach enabled a stratified assessment of the causal chain linking metabolic diseases, plasma metabolites, and CRC, with the aim of identifying shared metabolic mediators across various metabolic disorders. By elucidating this shared mechanism, this study provides universal intervention targets for populations with a high risk of CRC with coexisting metabolic diseases and provides novel insights into early disease prevention strategies.

## 2. Materials and Methods

### 2.1. Study Design

**Figure 1:**
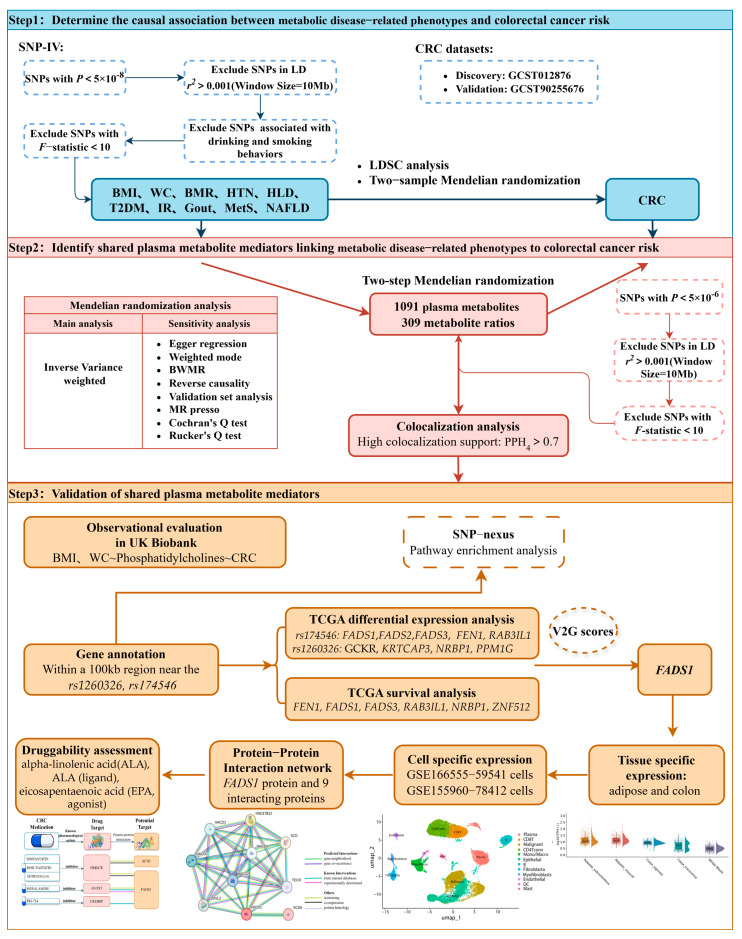
Flowchart of the study design. SNPs, single-nucleotide polymorphisms; CRC, colorectal cancer; BMI, body mass index; WC, waist circumference; BMR, basal metabolic rate; HTN, hypertension; HLD, hyperlipidemia; T2DM, type 2 diabetes mellitus; IR, insulin resistance; MetS, metabolic syndrome; NAFLD, non-alcoholic fatty liver disease; LDSC, linkage disequilibrium score; BWMR, Bayesian weighted Mendelian randomization; TCGA, The Cancer Genome Atlas.

This study systematically elucidates the causal pathway linking metabolic diseases, plasma metabolites, and CRC, using a TSMR mediation model. As illustrated in Figure 1, we first evaluated the genome-wide genetic correlation between CRC and 10 metabolic disease-related phenotypes—including BMI, waist circumference (WC), basal metabolic rate (BMR), hypertension (HTN), hyperlipidemia (HLD), T2DM, IR, gout, MetS, and NAFLD—through linkage disequilibrium score regression (LDSC), based on GWAS data from European populations. Subsequently, a TSMR analysis framework was used to screen for metabolic disease-related phenotypes with significant causal associations with CRC risk. Based on this, a two-step causal mediation model was constructed to identify shared plasma metabolite mediators: in the first step, metabolic disease-related phenotypes were used as exposures and plasma metabolites as outcomes to identify the metabolite candidates involved in metabolic regulation; and in the second step, the shared metabolites were used as exposures and CRC as the outcome to establish a cross-disease mediation pathway. To enhance the robustness of the results, we validated the metabolite-CRC associations in an independent CRC GWAS dataset, and the shared pathogenic variants of metabolites and CRC within the same chromosomal region were further considered by Bayesian colocalization. We further quantified the mediating effects and the proportion mediated by plasma metabolites. In addition, the correlation of phosphatidylcholine (PC) levels with BMI, WC and CRC incidence was validated in a UK Biobank (UKB) European cohort. At the molecular level, we performed a gene annotation and pathway enrichment analysis on single-nucleotide polymorphisms (SNPs) causally associated with both metabolites and CRC. Using RNA-seq data and clinical prognosis data from The Cancer Genome Atlas (TCGA), we evaluated the differential expression and prognostic significance of key genes, such as *FADS1*. Tissue-specific expression and cell type-specific analysis at the single-cell level were further carried out for *FADS1*. Finally, with the help of a protein–protein interaction (PPI) network and drug target database, the high-confidence interaction proteins of *FADS1* and their potential intervention targets were explored, which provided a systematic molecular basis for the early prevention and precise treatment of metabolic disease-associated CRC.

### 2.2. Data Sources

The information used for exposure, mediation and outcome in this study was mainly derived from pooled data from the GWAS conducted on individuals of European origin (Table S1).

#### 2.2.1. Exposure Data

A variety of metabolic disease-related indicators were included in this study, including core indicators of obesity (BMI, WC), thyroid function-related indicators (BMR), HTN, HLD, T2DM, IR, gout, MetS, and NAFLD, with sample sizes ranging from 37,037 to 468,298 individuals of European descent. Of these, pooled data on BMI, WC and BMR were obtained from the UKB European population cohort, with sample sizes of 461,460, 462,166, and 454,874 individuals, respectively, and with measurements taken by self-reporting or laboratory/healthcare facility collection, as well as genome-wide association analyses completed through the Medical Research Council Integrative Epidemiology Unit (MRC IEU)Open GWAS project [16]. Data were processed using age, age squared, sex and study site corrected residuals standardized to standard deviation (SD = 1) by normal transformed inverted order. Genetic data on HTN, HLD, and T2DM were obtained from the GWAS Catalog database (https://www.ebi.ac.uk/gwas/, accessed on 25 October 2024), and the corresponding GWAS analyses were carried out based on samples of 455,303, 349,222, and 468,298 cases from European populations. Indicators of IR (HOMA-IR) were calculated based on the MAGIC Consortium GWAS meta-analysis, applying a steady-state model assessment by fasting glucose and insulin levels, and included a total of 337,037 individuals [17]. Genetic data for gout were also obtained from the GWAS Catalog database, which contains GWAS analyses of 456,348 individuals of European ancestry [18]. Genetic data on MetS were obtained from the Dutch CNCR database (https://ctg.cncr.nl/, accessed on 25 October 2024), based on GWAS analyses of 461,920 individuals of European descent, with a total of 235 associated genomic risk loci identified [19]. Data on NAFLD were summarized from the IEU GWAS project, which conducted case–control GWAS analyses based on the UKB dataset, with diagnostic criteria reflecting the clinical diagnostic codes defined by the latest international consensus guidelines, resulting in the inclusion of 377,988 individuals of European origin (including 4761 NAFLD patients and 373,227 healthy controls) [20].

#### 2.2.2. Mediator Data

The plasma metabolites association data used in this study were derived from a large population-based cohort metabolomics study, which is the most comprehensive analysis of plasma metabolites available, with 1091 plasma metabolites and 309 metabolite ratios systematically examined by ultra-high performance liquid chromatography-tandem mass spectrometry (UHPLC-MS/MS). The corresponding summary statistics are publicly available from the GWAS Catalog (https://www.ebi.ac.uk/gwas/, accessed on 25 October 2024) under accession numbers GCST90199621 to GCST90201020. The study identified 690 molecular trait associations across 248 genetic loci, and 143 metabolite ratio associations across 69 independent loci. By integrating metabolite-gene associations with gene expression data, 94 putative causal genes were further identified, associated with 109 metabolites and 48 metabolite ratios [21].

#### 2.2.3. Outcome Data

GWAS data for CRC were obtained from the GWAS Catalog using access number GCST012876. The dataset includes 26,554 individuals of European origin, of which 11,895 are cases and 14,659 are controls, with a total of 38,370,461 SNPs detected [22]. To increase the reliability of the results, the CRC GWAS data used in this study during the validation phase were obtained from a genome-wide association study meta-analysis of a dataset consisting of 78,473 cases and 107,143 controls, all of European origin [23]. All the data were collected in accordance with ethical norms; subjects signed an informed consent form and the study protocol was approved by the local institutional review board.

### 2.3. Selection of IVs

Genetic instruments are well suited as IVs for MR analyses due to their unique properties, which can be a single genetic variant or a combination of multiple variants [24]. A qualified instrumental variable needs to fulfill three core assumptions. First, it should be strongly associated with the exposure factor (the correlation assumption). Second, it should have no significant association with confounding variables that may affect the final outcome (the independence assumption). Finally, its effect on the outcome must be transmitted only through the exposure factor without a direct relationship (the exclusion restriction hypothesis) (Figure S1). To screen for genetic variants suitable for a MR analysis and ensure the validity of IVs, we applied stringent selection criteria and rigorous quality-control procedures; details are provided in the Supplementary Note S1 [25,26,27,28,29].

### 2.4. Statistical Analyses

#### 2.4.1. LDSC Analysis

In the field of resolving the shared genetic structure of complex human phenotypes, the LDSC method based on GWAS data is widely used in genetic epidemiology. This method not only reliably assesses the heritability of traits by quantifying the chain imbalance effect among SNPs, but also systematically explores the genetic associations among different traits. In this study, we focused on the potential genetic associations between metabolic disease-related phenotypes and the risk of CRC. During the analysis, we constructed a reference linkage disequilibrium (LD)panel using European population data from the 1000 Genomes Project (https://github.com/bulik/ldsc, accessed on 25 October 2024), to adjust for correlations between genetic variants. According to the study design, the statistical significance threshold was set at *α* = 8.33 × 10^−3^ (0.05/6) after correction for Bonferroni’s multiple testing, and significant genetic associations were determined when the *p*-value obtained from the test was below this threshold.

#### 2.4.2. TSMR Analysis

TSMR was used in this study to identify significant associations between (1) metabolic disease-related phenotypes and CRC risk, (2) metabolic disease-related phenotypes and plasma metabolites, and (3) plasma metabolites co-associated with metabolic disease-related phenotypes and CRC. The core methodology of this study used the inverse variance weighted (IVW) approach to construct an overall causal estimate by aggregating the SNP-specific effect sizes using a weighted average. The IVW approach assumes the presence of balanced pleiotropy and employs a multiplicative random-effects model to provide consistent estimates of the causal effects [30]. To validate the robustness of the results, the study simultaneously applied three complementary approaches: (1) MR-Egger regression, which introduces the InSIDE assumption (instrument strength independent of direct effect) and uses the intercept to detect directional pleiotropy; the slope provides the pleiotropy-adjusted causal estimate [31]; (2) weighted median, which yields a consistent causal estimate under the assumption that at least 50% of the total weight comes from valid instruments, and remains robust even if a proportion (less than 50%) of the instruments is invalid [32]; (3) Bayesian weighted Mendelian randomization (BWMR), which automatically identifies anomalies by constructing hierarchical probabilistic models and using a posteriori probability weighting to automatically identify abnormal SNPs, thus effectively controlling the effects of multigene background noise and horizontal pleiotropy on causal estimation [33]. In this study, we used the “*TwoSampleMR*” v0.5.6 R package and “*BWMR*” v0.1.1 R package to perform the above MR analysis. In identifying plasma metabolites causally associated with metabolic disease-related phenotypes, the Benjamini–Hochberg method was used for false discovery rate (FDR) correction, and metabolites for which both the primary method and BWMR method were significant (FDR-adjusted *p*-values, *p*_fdr_ < 0.05) were selected to ensure credibility.

#### 2.4.3. Sensitivity Analysis

Based on the results of the MR analyses described above, a series of further multivariate sensitivity analyses were conducted in this study to identify the core causal inference methods. The presence of horizontal pleiotropy was first detected by the MR-PRESSO global test [34]. Then, MR-Egger regression was used to assess potential directional pleiotropy based on the intercept term. At the same time, the heterogeneity of the IVW model was assessed by calculating Cochran’s Q statistic and quantifying the residual variance of the MR-Egger regression by applying Rucker’s Q metric. The criteria for selecting the final causal inference method are shown in Supplementary Note S2.

In addition, to ensure the robustness of the results, significant results were analyzed in this study using the leave-one-out method, which excludes each instrumental variable one by one and re-estimates the overall causal effect after the exclusion to confirm that individual IVs did not abnormally drive the overall results. Meanwhile, to further assess the directionality of associations, we performed reverse MR analyses. These analyses examined whether shared plasma metabolites associated with CRC could in turn affect metabolic disease-related phenotypes, and whether CRC itself influenced the identified shared plasma metabolites. This approach comprehensively confirmed the triangular causal relationships among the three traits. Ultimately, the relevant shared plasma metabolites identified in the discovery set were further validated in the CRC validation set to ensure the reliability and authenticity of the MR analysis.

#### 2.4.4. Colocalization Analysis

Although MR can identify causal relationships between two phenotypes, they may be driven by different genetic variants. To explore the potential impact of this discrepancy on MR estimation, we performed colocalization analyses to assess whether the SNP instruments for metabolites and the GWAS signals for CRC share the same causal variants. Detailed methods and threshold settings for the colocalization analyses are provided in Supplementary Note S3 [35,36].

#### 2.4.5. Mediator Analysis

In this study, we used a two-step MR approach for mediation effect analysis. Specifically, the first stage MR quantified the causal effects of metabolic disease-related phenotypes and plasma metabolites $\beta_{1}$, and the second stage MR assessed the independent causal effects of the target metabolites on the risk of CRC $\beta_{2}$, and the IVs used in the second stage should exclude the IVs used in the first stage. Meanwhile, the total effect estimates of metabolic disease-related phenotypes and CRC were obtained directly by TSMR analysis $\beta_{3}$. Based on the coefficient product method, the mediation effect was calculated as $\beta_{1}\times \beta_{2}$ and the mediation proportion as $(\beta_{1}\times \beta_{2})/\beta_{3}$ [37]. To assess the statistical stability of the estimates, the study used parametric analysis (Delta method) to derive the asymptotic standard errors of the mediated effect sizes and constructed 95% confidence intervals for statistical inference [38].

#### 2.4.6. Observational Analysis

In this study, multiple linear regression analyses were used to assess the association between baseline BMI, WC and plasma PC levels in 265,603 individuals from the UKB. In this study, multiple linear regression analyses were performed to examine the association between baseline BMI, WC, and plasma PC levels among 265,603 participants from the UKB. Multivariable Cox proportional hazards regression analysis was also used to evaluate associations between baseline plasma PC levels and CRC risk. Cohort details and methodological procedures are provided in Supplementary Note S4.

#### 2.4.7. Gene Annotation, Differential Expression Analysis and Survival Analysis

In order to delve deeper into the association between intermediary metabolites and CRC at the gene level, we first performed a systematic gene annotation analysis of all the genes located within the 100 kb region around the shared causal SNPs. This step was aimed at identifying more candidate genes associated with this SNP and determining the functional genes that are most likely to be regulated by it, thus revealing the potential mechanism of action of this genetic variant in regulating metabolite levels and carcinogenesis. We conducted a pathway enrichment analysis for the identified causal SNPs using the SNPnexus platform (https://www.snp-nexus.org, accessed on 6 December 2024). We conducted differential expression and survival analyses on the candidate functional genes using RNA-seq data from the TCGA-COAD cohort (colorectal adenocarcinoma, *n* = 524). Differential expression analysis was performed using the “*DESeq2*” v.1.34.0 package to perform variance stabilizing transformation (VST) on the raw count data. For each selected gene, boxplots were generated and the Mann–Whitney–Wilcoxon rank-sum test was used to compare VST-normalized expression values between tumor and normal tissue. Genes with *p*_fdr_ < 0.05 were considered significantly differentially expressed in CRC. For the survival analysis, we applied univariate Cox proportional hazards regression to evaluate the association between overall survival (OS) and the expression levels of 14 genes located near the shared causal SNPs. The optimal expression cutoff points for each gene were determined using “*survminer*” v.0.5.0, and the samples were divided into high- and low-expression groups. Kaplan–Meier survival curves were plotted, and genes with *p*_fdr_ < 0.05 were defined as prognostically relevant. Finally, the genes with both differential expression and prognostic significance were incorporated into a multivariable Cox regression model to derive the regression coefficients for each gene. An individualized risk score model was then constructed based on the formula

(1) $$Risk\;Score=\sum_{i=1}^{n}\beta_{i}\times Exp_{i}$$

where n denotes the number of genes, i denotes the ith gene, $\beta_{i}$ denotes the regression coefficient of the ith gene, and $Exp_{i}$ denotes the expression level of the ith gene. Subsequently, using the optimal cutoff value of the risk score, patients were stratified into high-and low-risk groups, and Kaplan–Meier survival analysis was performed to assess the prognostic discrimination ability of the model.

#### 2.4.8. Tissue-Specific Expression Analysis

In this study, the *rs174546* locus was queried in the Open Targets Genetics database [39] to evaluate its variant-to-gene (V2G) score and identify its most likely target gene. Subsequently, bulk RNA-seq expression data from 5 tissues—subcutaneous adipose tissue, visceral adipose tissue, transverse colon, sigmoid colon, and whole blood—were downloaded from the GTEx Portal (https://gtexportal.org/, accessed on 17 December 2024) [40]. Violin plots were generated to visualize the expression levels of *FADS1* across the five tissues. To compare tissue-specific differences, two-sided Wilcoxon rank-sum tests were performed between *FADS1* expression in whole blood and that in each of four other tissues (subcutaneous adipose, visceral adipose, transverse colon, and sigmoid colon).

#### 2.4.9. Single-Cell RNA Sequencing Analysis

To investigate *FADS1* expression at single-cell resolution in adipose tissue and the colorectum, we reanalyzed gene expression matrix data from the GSE155960 dataset in the Gene Expression Omnibus (GEO) and the GSE166555 dataset in the Tumor Immune Single-cell Hub (TISCH), focusing on *FADS1* expression in adipose and tumor tissues [41]. The GSE166555 dataset consists of tissue samples collected from the tumor area and adjacent normal tissues of 12 CRC patients [42], while the GSE155960 dataset comprises single-cell RNA sequencing data from three lean and three obese individuals [43]. During the initial quality control of the raw gene expression matrices, cells with fewer than 600 or more than 4000 detected genes were excluded, as well as those with fewer than 1000 total UMIs, to eliminate low-quality cells and potential doublets. The retained cells were normalized by library size, and the top 2000 highly variable genes were identified using the FindVariableFeatures function in the “*Seurat*” v5.1.0 R package for downstream dimensionality reduction. After centering and scaling these 2000 genes, a nearest-neighbor graph (FindNeighbors) was constructed based on the top 10 principal components, and cell clusters were identified by Louvain’s algorithm (FindClusters) using default parameters. Subsequently, uniform manifold approximation and projection (UMAP) was used to visualize the clusters in 2 dimensions, and clusters were manually annotated based on known marker genes to determine the cell types represented by each cluster.

To test whether *FADS1* was significantly enriched in specific cell types, we calculated the proportion of *FADS1*-positive cells (defined as non-zero expression) within each annotated cell type. A label permutation test was performed with 1000 iterations to generate a null distribution of *FADS1*-positive proportions. The permutation *p*-value was calculated as the frequency with which the permuted datasets produced proportions equal to or exceeding the observed value, providing a non-parametric statistical measure of *FADS1* expression enrichment across cell types.

#### 2.4.10. PPI Network, Enrichment Analysis and Druggability Assessment

PPI network analysis was performed using the STRING database (https://string-db.org/, accessed on 30 December 2024) to identify proteins interacting with *FADS1* and assess their interactions with approved drug targets for CRC. Gene ontology (GO) and Kyoto Encyclopedia of Genes and Genomes (KEGG) pathway enrichment analyses were performed using the Database for Annotation, Visualization and Integrated Discovery (DAVID) [44] to characterize the biological processes and pathways enriched among these interacting proteins. To assess the druggability of proteins in the *FADS1*-associated interaction network, we queried multiple drug-target databases, including DrugBank [45], Open Targets and the Therapeutic Targets database [46].

## 3. Results

### 3.1. Causal Estimation of Metabolic Disease-Related Phenotypes for CRC

The heritability h^2^ of the 10 metabolic disease-related phenotypes is shown in Table 1. The heritability contribution of BMI, WC, BMR, and HTN was high, with HTN having a heritability contribution of 0.885, while HLD, T2DM, IR, gout and NAFLD had significantly low heritability. Further bivariate analysis by LDSC showed that BMI, WC, BMR, IR and MetS were all significantly positively genetically associated with CRC, with IR showing the strongest correlation (rg = 0.473, *p* = 8.61 × 10^−4^), followed by MetS (rg = 0.197, *p* = 8.69 × 10^−6^), WC (rg = 0.156, *p* = 8.62 × 10^−5^), and BMI (rg = 0.126, *p* = 3.28 × 10^−3^). Therefore, HTN, HLD, T2DM, gout, and NAFLD, which are not genetically associated with CRC, were excluded from subsequent analyses.

Based on the GWAS datasets, this study screened SNPs as IVs for five metabolic disease-related phenotypes (BMI, WC, BMR, IR and MetS). Among them, 458, 374, 8, 546, and 193 IVs were obtained by genomic significance threshold screening for BMI, WC, IR, BMR, and MetS, respectively. The cumulative variance (R^2^) explained by these instruments was 6.32%, 4.63%, 0.49%, 9.61%, and 2.27%, respectively. The F-statistics of all the selected SNPs were greater than 20, indicating that the selected IVs were sufficiently potent and the risk of weak instrumental bias was extremely low. More information about these selected IVs is detailed in Table S2.

**Figure 2:**
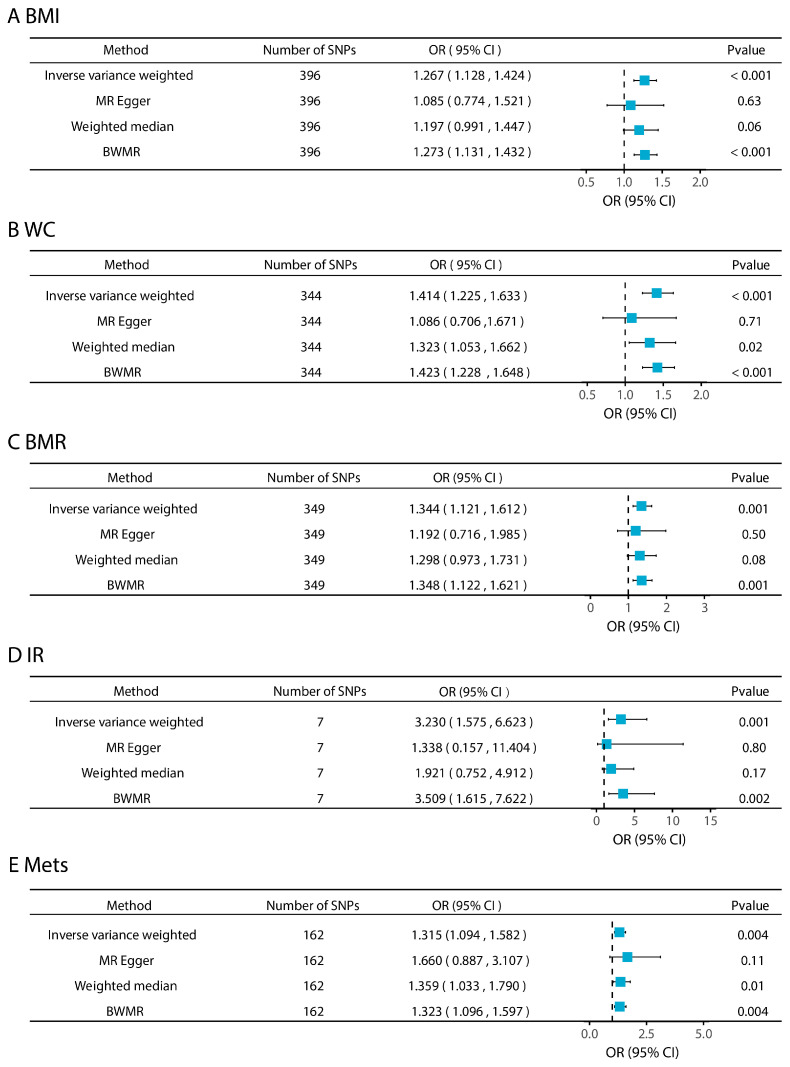
Effects of metabolic disease-related phenotypes on colorectal cancer.

By the two-sample MR analysis, this study assessed the causal associations between genetically predicted levels of five metabolic disease-related phenotypes and CRC risk (Figure 2). The SNVs included in each analysis are listed in Tables S3–S7. The main analysis using the IVW method revealed significant positive associations between genetically predicted increases in BMI (OR = 1.267, 95% CI: 1.128–1.424, *p* < 0.001), WC (OR = 1.414, 95% CI: 1.225–1.633, *p* < 0.001), BMR (OR = 1.344, 95% CI: 1.121–1.612, *p* = 0.001), IR (OR = 3.230, 95% CI: 1.575–6.623, *p* = 0.001), and MetS (OR = 1.315, 95% CI: 1.094–1.582, *p* = 0.001) and increased CRC risk. Sensitivity analyses further supported the robustness of the results: the F-statistics of all the SNPs associated with these exposures were greater than 10 (Table S8), suggesting a low risk of bias for the weak IVs. No evidence of horizontal pleiotropy was detected based on MR-PRESSO global tests (*p* > 0.05) or MR-Egger intercepts (intercept *p* > 0.05; Table S9). The scatter plot visualizes the SNP-specific associations with metabolic disease-related phenotypes and CRC risk, supporting the assumption of a linear causal relationship (Figure S2). Leave-one-out analyses showed that causal estimates were not driven by a single SNP (Figure S3). The statistical power analysis showed that, given the sample size and effect sizes observed, the study was sufficiently powered to detect causal effects for all five phenotypes (Table S10).

### 3.2. Effects of Five Metabolic Disease-Related Phenotypes on Plasma Metabolites

**Figure 3:**
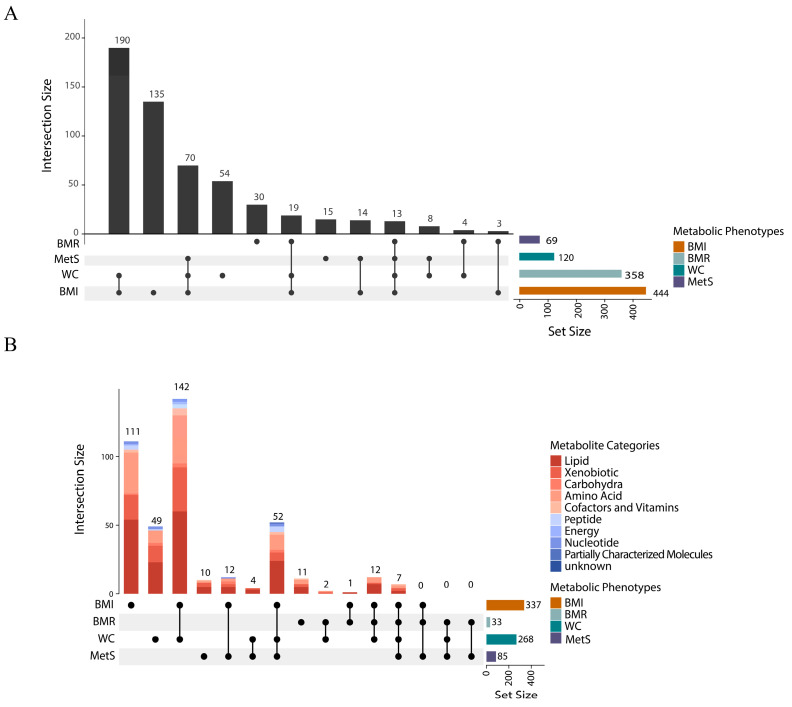
Plasma metabolites associated with four metabolic disease-related phenotypes. (**A**). UpSet diagram of the number of plasma metabolites (1091 metabolites and 409 metabolite ratios) associated with metabolic disease-related phenotypes. (**B**). UpSet diagram of 555 metabolites associated with at least one metabolic disease-related phenotype. The horizontal bar on the right represents the number of set size in each covariate. Dots and lines represent subsets of set size. The vertical histogram represents the number of set size in each subset.

We performed MR analyses of five CRC-related metabolic disease-related phenotypes on 1091 plasma metabolites and 309 metabolite ratios. After FDR correction and combined with the BWMR method, the two-sample MR analysis revealed that four metabolic disease-related phenotypes (excluding IR) exhibited significant causal effects on plasma metabolites and ratios (Figure 3A,B). Specific detailed MR results are shown in Tables S11 and S12.

These causal relationships were further visualized using a circular heatmap (Figures S4–S7), demonstrating metabolite associations across multiple functional categories, including lipids, amino acids, xenobiotics, carbohydrates, cofactors and vitamins, peptides, energy metabolites, nucleotides, and partially characteristic molecules. Lipid metabolites have the highest percentage among all categories. A total of 337 plasma metabolites and 107 metabolite ratios were identified as causally associated with BMI (*p*_fdr_ < 0.05), with lipid metabolites accounting for a substantial proportion. An analysis of the key metabolites showed that the top five metabolites most significantly associated with BMI were fructose metabolism-related mannose (IVW *β* = 0.413, 95% CI: 0.322–0.505, *p*_fdr_ = 5.45 × 10^−16^), carotenoid from the cofactors and vitamins group (IVW *β* = 0.093, 95% CI: 0.000–0.186, *p*_fd_ = 1.64 × 10^−15^), mannonate (IVW *β* = 0.368, 95% CI: 0.277–0.461, *p*_fd_ = 5.64 × 10^−13^), and hydroxyasparagine (IVW *β* = 0.316, 95% CI: 0.236–0.396, *p*_fd_ = 1.20 × 10^−12^). The top five significant metabolites were mainly related to glucose metabolism and vitamin pathways, and the next highest were all lipid metabolites. In the BMR-associated group, the top five metabolites were highly similar to those for BMI, including mannose, carotene, mannonate, hydroxyasparagine, and carotene diol. In the WC-associated group, the top metabolites were dominated by amino acid and sugar metabolites such as hydroxyasparagine and mannose, along with the lipid-specific molecule 1-(1-enyl-palmitoyl)-2-oleoyl-GPC. For MetS, the top five most significantly associated metabolites included two lipids (1-(1-enyl-palmitoyl)-2-oleoyl-GPC and cortolone glucuronide), one nucleotide (N4-acetylcytidine), and fructose metabolism-related mannose and mannonate. In contrast, no significantly associated metabolites were found for IR.

Of four metabolic phenotypes, seven metabolites and six metabolite ratios showed common correlations (Table S13); the seven metabolites were mannose, mannonate, hydroxyaspartate, N2, n2-dimethylguanosine, N4-acetylcytidine, 3*β*-hydroxy-5-cholestenoate and glycerophosphoethanolamine. Furthermore, obesity-related metabolic phenotypes (BMI, WC) and MetS had 59 co-associated metabolites and 24 co-associated metabolite ratios. The obesity-related metabolic phenotypes (BMI, WC) and BMR had 19 co-associated metabolites and 13 co-associated metabolite ratios.

The results of the polygenic and heterogeneity tests for plasma metabolite SNPs are shown in Table S14. All of the above MR results passed the Cochran’s Q test (*p* > 0.05), suggesting limited heterogeneity among IVs. According to pre-defined criteria, IVW was used as the primary estimation method when both MR-PRESSO and MR-Egger tests indicated no evidence of horizontal pleiotropy. Otherwise, alternative MR methods were selected as the main result to ensure robustness.

### 3.3. Effect of Intermediate Metabolites on CRC

**Figure 4:**
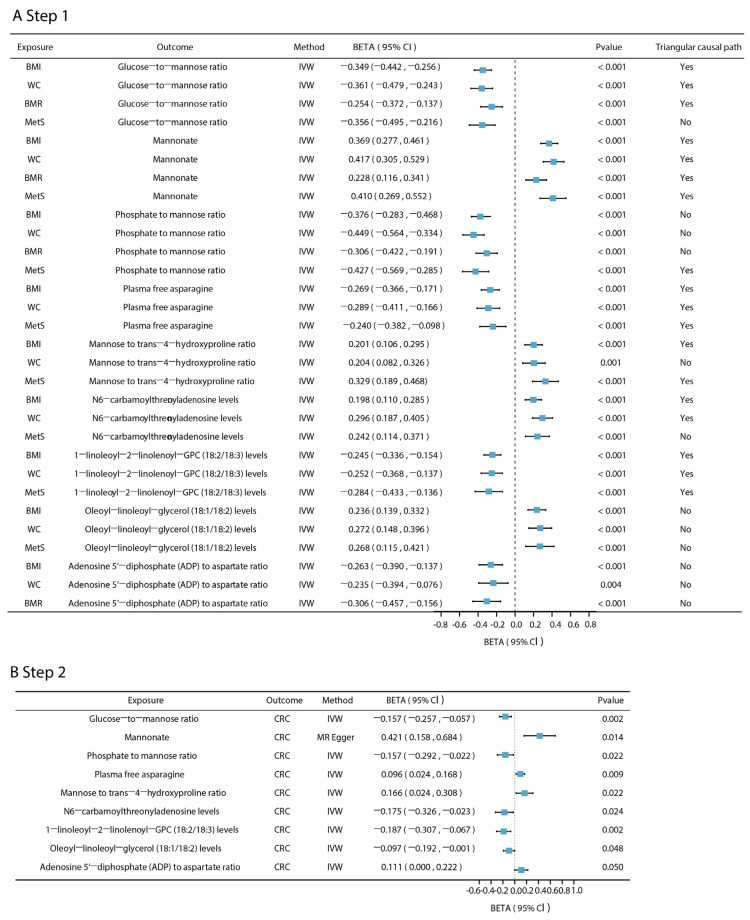
Two-step MR results for mediated plasma metabolites. (**A**). The effect of five phenotypes associated with CRC on plasma metabolites. (**B**). The effect of plasma metabolites and metabolite ratios on CRC. The *P*_fdr_ of the above results are all less than 0.05 (*p*_fdr_ < 0.05). Triangular causal path: the triangular causal paths formed by metabolic disease-related phenotypes, plasma metabolites, and CRC. “Yes” means that the proportion of intermediaries is significant and there is no reverse causality in the triangular causal path; “No” means that the mediation effect is not significant or that there is reverse causality in the triangular causal path.

Across the metabolome-wide landscape, we applied a two-step MR approach using four metabolic disease-related phenotypes—BMI, WC, BMR, MetS—to identify plasma metabolites and metabolite ratios that were jointly associated with at least two of four phenotypes (Figure 4A excluding those associated with BMI and WC only). These metabolites were then further evaluated for their potential causal relationships with CRC. The *F*-statistics for all metabolite IVs exceeded 10, indicating strong instrument strength (Table S15). Among the seven shared metabolites and six shared metabolite ratios that were co-related to four phenotypes, one metabolite and two metabolite ratios were found to be causally associated with CRC (Figure 4B): the glucose–to–mannose ratio (*β* = −0.157, 95% CI: −0.257–−0.057, *p* = 0.002), the xenobiotic mannonate (*β* = 0.421, 95% CI: 0.158–0.684, *p* = 0.014), and the phosphate–to–mannose ratio (*β* = −0.157, 95% CI: −0.292–−0.022, *p* = 0.022). Notably, the same SNP locus, *rs1260326*, was present in the IVs for all three plasma metabolites. Of the 83 co-associated metabolites shared by obesity-associated metabolic phenotypes (BMI, WC) and MetS, in addition to the three metabolites mentioned above, the plasma free asparagine involved in the metabolism of glutamic acid and aspartic acid was causally associated with CRC (*β* = 0.096, 95% CI: 0.024–0.168, *p* = 0.009), as were the N6-carbamoylthreonyladenosine involved in purine metabolism (*β* = −0.175, 95% CI: −0.326–−0.023, *p* = 0.024); lipids in the form of 1-linoleoyl-2-linolenoyl-GPC (18:2/18:3) (*β* = −0.187, 95% CI: −0.307–−0.067, *p* = 0.002) and oleoyl–linoleoyl–glycerol (18:1/18:2) (*β* = −0.097, 95% CI: −0.192–−0.001, *p* = 0.048); the mannose–to–trans-4-hydroxyproline ratio (*β* = 0.166, 95% CI: 0.024–0.308, *p* = 0.022); and the phosphate-to-mannose ratio (*β* = −0.157, 95% CI: −0.293–−0.023, *p* = 0.022). The adenosine 5′-diphosphate (ADP)–to–aspartate ratio was among the metabolic metabolites co-associated with obesity-related metabolic phenotypes (BMI, WC), and BMR was causally associated with CRC (*β* = 0.111, 95% CI: 0.000–0.221, *p* < 0.05) (Table S16). In addition, Cochran’s Q-test indicated no significant heterogeneity among IVs. With the exception of mannonate, which failed the MR-Egger intercept test (*p* = 0.042 < 0.05) and for which MR-Egger was used as the primary method, all other metabolites passed both the MR-PRESSO and MR-Egger tests, indicating no evidence of horizontal pleiotropy (Table S16). The statistical power for detecting causal effects under the given sample sizes and effect estimates is provided in Table S17. A leave-one-out analysis further confirmed the robustness of the main findings, showing no single SNP disproportionately influenced the results (Figure S8).

To validate the hypothesized triangular causal pathway in which metabolic disease-related phenotypes influence CRC via plasma metabolites, we ruled out any potential reverse causality by bidirectional MR. The phosphate–to–mannose ratio was found to be reverse-causally associated with BMI (*β* = 0.036, 95% CI: 0.023–0.050), WC (*β* = 0.027, 95% CI: 0.015–0.040), and BMR (*β* = 0.008, 95% CI: 0.062–0.098) (all *p* < 0.001). Similarly, the glucose–to–mannose ratio exhibited reverse causality with MetS (*β* = 0.124, 95% CI: 0.066–0.182, *p* < 0.001) (Table S18). These findings should be regarded as exploratory and warrant validation in independent datasets. Consequently, to avoid confounding the triangular causal pathway analysis, these metabolite-metabolic phenotype causal pairs with reverse causality were excluded.

To further validate the causal network, we repeated the MR analysis using an independent CRC GWAS dataset. All four metabolic disease-related phenotypes retained statistically significant causal associations with CRC (Table S19). The glucose–to–mannose ratio, mannonate levels, 1-linoleoyl-2-linolenoyl-GPC (18:2/18:3) and mannose–to–trans-4-hydroxyproline ratio remained significantly associated with CRC. While other metabolites did not reach statistical significance, their directions of effect were consistent with previous findings, suggesting potential causal roles. For MR-significant metabolites, detailed heterogeneity and pleiotropy test results are provided in Table S20. Due to the moderate evidence of pleiotropy and heterogeneity for the mannose–to–trans-4-hydroxyproline ratio and 1-linoleoyl-2-linolenoyl-GPC (18:2/18:3), we adopted weighted MR models as the primary method for inference.

To support the mediation hypothesis, we performed a colocalization analysis between plasma metabolites and CRC. As shown in Table S21, 1-linolenoyl-2-linolenoyl-GPC (18:2/18:3), a metabolite jointly associated with obesity and MetS, had a posterior probability of H4 of 86.1% within a 500 kb window, suggesting that *rs174546* is likely a shared causal variant. The colocalization plot for *rs174546* is provided in Figure S9. In addition, the posterior probability of H_4_ at the oleoyl–linoleoyl–glycerol (18:1/18:2) level was 64.1%, indicating potential shared causal genetic variation with CRC. For the adenosine 5′-diphosphate (ADP)–to–aspartate ratio, a metabolite jointly associated with obesity and BMR, the PPH_4_ was 70.8%, suggesting that *rs35540647* may be a shared causal variant for both the metabolite and CRC risk.

Collectively, these findings provide evidence supporting the hypothesis that plasma metabolites may act as potential mediators in the causal pathway linking metabolic disease-related phenotypes and CRC.

### 3.4. Mediating Effects of Plasma Metabolites in the Association Between Metabolic Disease-Related Phenotypes and CRC Risk

**Figure 5:**
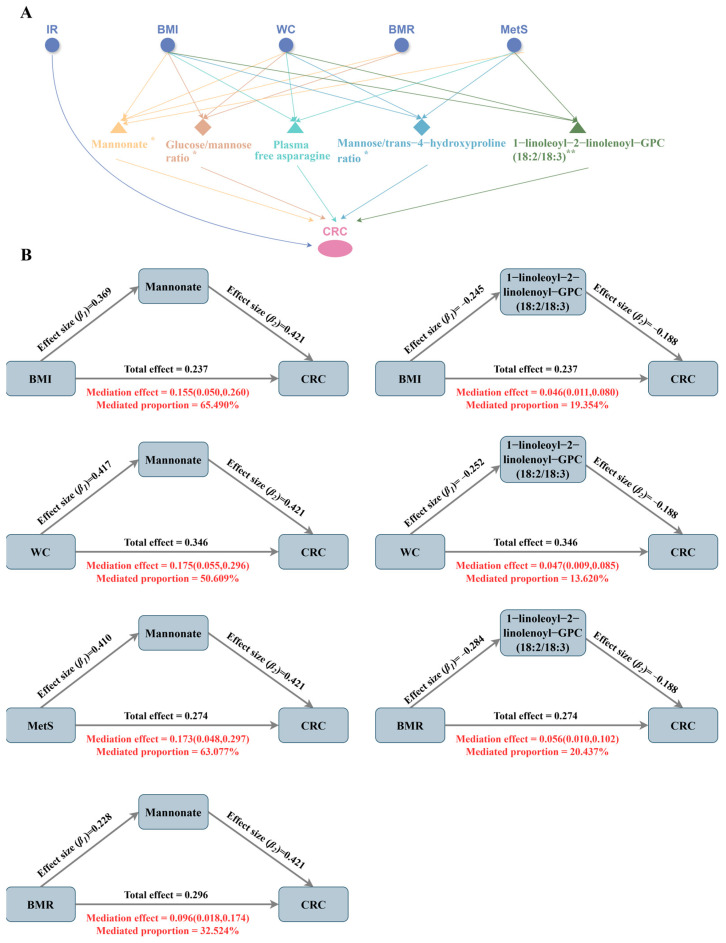
Common intermediary metabolites between metabolic phenotypes and CRC. (**A**). ‘metabolic phenotype–plasma metabolites–CRC’ triangular causal network; ** indicates the highest level of evidence for this metabolite, i.e., it has passed colocalization analysis and has a significant causal relationship in the external validation set; * indicates that the evidence level for this metabolite is secondary, i.e., it has passed colocalization analysis or has a significant causal relationship in the external validation set. (**B**). Mediating ratios of the mannonate levels and 1-linoleoyl-2-linolenoyl-GPC (18:2/18:3) levels in the causal pathway.

As shown in Figure 5 (see Table S22 for specific values), Figure 5A illustrates the constructed “metabolic phenotype–plasma metabolites–CRC” triangular causal network, revealing the potential bridging roles of several shared mediating metabolites across different pathways. Figure 5B presents the estimated mediation proportions of each metabolite in the respective pathways. To highlight the key mediators, only the triangular causal pathway formed by mannonate and 1-linolenoyl-2-linolenoyl-GPC (18:2/18:3) is shown, and mediation effects for other metabolites are provided in Figure S10. Mannonate played a central role in the causal pathways of all four metabolic phenotypes in CRC, with mediation proportions of 65.490% for BMI, 50.609% for WC, 63.077% for BMR, and 32.524% for MetS. The glucose–to–mannose ratio was a common mediator of BMI, WC and BMR, mediating 23.155%, 16.397% and 13.526%, respectively. In addition, plasma free asparagine and 1-linolenoyl-2-linolenoyl-GPC (18:2/18:3) co-mediated the causal effects of BMI, WC and MetS on CRC, with the mediating proportions of asparagine at 10.970%, 10.991%, and 7.774%, respectively, while the mediated ratios of 1-linolenoyl-2-linolenoyl-GPC (18:2/18:3) were higher, at 19.354%, 13.620% and 20.437%, respectively. Although the phosphate–to–mannose ratio showed reverse causal associations with BMI, WC, and BMR, it was found to specifically mediate the effect of MetS on CRC, with a mediation proportion of 24.546%. The mannose–to–trans-4-hydroxyproline ratio served as a specific mediator for BMI (14.048%) and MetS (19.894%). In contrast, the mediating effects of oleoyl–linoleoyl–glycerol (18:1/18:2) levels and the adenosine 5′-diphosphate (ADP)–to–aspartate ratio did not reach statistical significance (*p* > 0.05), possibly due to the limited statistical power arising from a small number of valid genetic instruments.

### 3.5. Association Between BMI and WC and Plasma PC Levels in UK Biobank

1-linolenoyl-2-linolenoyl-GPC (18:2/18:3), a subclass of PC, was identified in this study as a mediator of the causal effects of BMI, WC and MetS on CRC. Given that the UKB database provides only total PC level measurements, we further investigated the associations between BMI, WC, and plasma PC levels to validate this link. Multiple linear regression models were constructed to assess the effect of baseline BMI and WC on plasma PC levels in 265,603 participants from the UKB. The models were simultaneously adjusted for age, sex, recruitment center, metabolite measurement batch and sample processing delay time to ensure that the results were not confounded by these potential confounders. As shown in Table 2 (see also Table S23), after covariate adjustment, BMI was significantly negatively associated with plasma PC levels (*β* = −0.020, 95% CI: −0.021–−0.019, *p* < 0.001); and WC was significantly associated with decreased plasma PC levels (*β* = −0.008, 95% CI: −0.008–−0.007, *p* < 0.001). When BMI and WC were included simultaneously in the model, both retained independent and significant inverse associations with plasma PC levels, with effect estimates of *β* = −0.015 (*p* < 0.001) for BMI and *β* = −0.003 (*p* < 0.001) for WC.

### 3.6. Association Between Plasma PC Levels and CRC Incidence in the UK Biobank

Multivariate Cox proportional risk regression analyses were performed in order to assess the association between plasma PC levels and the risk of CRC incidence. A total of 273,765 participants from the UKB were included, comprising 11,867 incident CRC cases and 261,898 controls. Time to event was calculated as “date of onset (or last follow-up)–date of enrolment”, with a focus on events occurring within 15 years of enrolment. Individuals with a CRC diagnosis prior to baseline or missing event dates were excluded.

Due to the large variation in sample size among recruitment centers, we used “recruitment center” as a stratification variable in the model, and adjusted for covariates such as age, sex, BMI, metabolite measurement batch, and sample processing delay time. As shown in Table 3 (see also Table S23), the risk of CRC was significantly higher with each standard deviation increase in PC levels (HR = 1.21, 95% CI: 1.15–1.27, *p* = 3.61 × 10^−13^). An analysis stratified by sex revealed that this association was more pronounced in males (HR = 1.34, 95% CI: 1.25–1.44, *p* = 2.33 × 10^−16^), whereas no significant association was observed in females. These findings are consistent with the results from a nested case–control study in a U.S. population cohort [47].

### 3.7. Gene Annotation: Molecular Associations Between Plasma Metabolite Mediators and CRC

**Figure 6:**
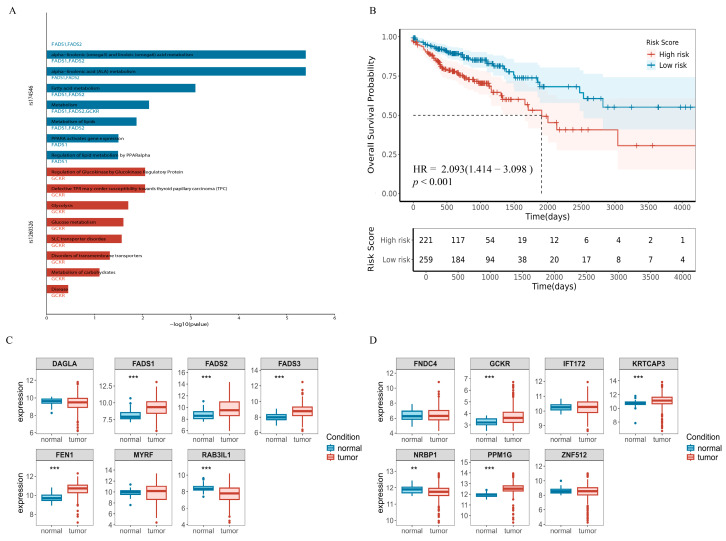
Molecular associations between plasma metabolite mediators and CRC. (**A**). Plot of results of a pathway enrichment analysis of causal SNP loci in the SNPnexus website. (**B**). Survival curves for the five best prognostic gene combinations. HR and confidence intervals from Cox analysis, *p*-values from log-rank test. (**C**). Boxplot of gene expression differences near *rs174546*. (**D**). Gene expression differences near *rs1260326*. Vertical coordinates represent the distribution of miRNA expression (raw count data were variance stabilized and transformed using *DESeq2*). Box plots display the median (center line), 25th and 75th percentiles (box bounds), (** *p* value < 0.01; *** *p* value < 0.001).

To further elucidate the potential molecular mechanisms by which the identified key mediating metabolites contribute to CRC development, we focused on metabolite mediators with the highest level of evidence and selected their corresponding shared pathogenic SNPs—*rs174546*, which colocalized with 1-linoleoyl-2-linolenoyl-GPC (18:2/18:3) and CRC, and *rs1260326*, a common instrumental variable for glucose–to–mannose ratio, mannonate, and phosphate–to–mannose ratio. We extracted candidate genes located within ± 100 kb of each SNP to identify potentially regulated functional targets. A total of 23 genes were finally identified, namely *DAGLA*, *MYRF*, *MYRF-AS1*, *LOC124902680*, *LOC124902679*, *FEN1*, *TNEM258*, *MIR611*, *FADS2*, *FADS1*, *FADS3*, *MIR1908*, *RAB3IL1*, *MIR6746*, *PPM1G*, *NRBP1*, *KRTCAP3*, *IFT172*, *RNU6-986P*, *FNDC4*, *GCKR*, *SPATA31H1*, *ZNF512*. A pathway enrichment analysis via SNPnexus revealed that the association between 1-linoleoyl-2-linolenoyl-GPC (18:2/18:3) and CRC is linked to several lipid metabolic pathways, including linoleic acid metabolism (*p* = 1.00 × 10^−6^), α-linolenic (omega 3) and linoleic (omega 6) acid metabolism (*p* = 4.00 × 10^−6^), α-linolenic acid (ALA) metabolism (*p* = 4.00 × 10^−6^), and fatty acid metabolism (*p* = 7.96 × 10^−4^) (Figure 6A, Table S24). The *rs1260326* was enriched in the regulation of glucokinase by the glucokinase regulatory proteins pathway (*p* = 1.18 × 10^−2^).

Next, we performed gene expression and survival analyses using RNA-seq data from TCGA, comprising 41 normal controls and 483 CRC tumor samples. We analyzed only 14 genes that all had non-zero expression in at least 80% of the samples (Figure 6C,D), and observed that genes located near *rs174546*—including *FADS1*, *FADS2*, *FADS3*, and *FEN1*—were significantly upregulated in CRC tissues compared to normal tissues (*p* = 1.5 × 10^−9^, 3.3 × 10^−6^, 2.80 × 10^−9^, and 5.90 × 10^−16^, respectively). While *RAB3IL1* was significantly downregulated (*p* = 1.5 × 10^−6^), for genes near *rs1260326*, GCKR (*p* = 4.6 × 10^−6^), *KRTCAP3* (*p* = 1.9 × 10^−5^), *NRBP1* (*p* = 6.5 × 10^−3^) and *PPM1G* (*p* = 2.0 × 10^−16^), genes were significantly differentially expressed in CRC, except for *NRBP1*, which showed upregulation in CRC. These differentially expressed genes may be closely associated with CRC development and progression, suggesting that these genes may play an important role in tumor formation through specific genetic mechanisms or regulatory networks.

A survival analysis was performed using univariate Cox proportional hazards regression as well as plotting Kaplan–Meier survival curves, and the results, as shown in Table S25 as well as Figures S11 and S12, revealed that six genes, *FEN1*, *FADS1*, *FADS3*, *RAB3IL1*, *NRBP1* and *ZNF512*, were significantly associated with overall survival in CRC patients (*p*_fdr_ < 0.05). Among them, *FEN1*, *FADS1*, *FADS3*, *RAB3IL1* and *NRBP1* genes fulfilled the dual criteria of differential expression and significant prognostic features, suggesting that five genes have a significant impact on the development and prognosis of CRC. Subsequently, the multivariate Cox regression model was performed to obtain the regression coefficients of the five best prognostic genes, and the expression levels and coefficients of the individual genes were combined using a linear combination to obtain the risk score formula:

Risk score = 0.04987 × Exp (*FEN1*) − 0.08195 × Exp (*FADS3*) + 0.04919 × Exp (*FADS1*) + 0.26857 × Exp (*RAB3IL1*) + 0.06046 × Exp (*NRBP1*) (2)

Patients were classified into high-risk (*n* = 245) and low-risk (*n* = 235) groups based on the optimal cutoff point. Kaplan–Meier survival curves showed that the high-risk group had significantly worse overall survival compared to the low-risk group (HR = 2.015, 95% CI:1.351–3.005, *p* = 5.0 × 10^−4^, Figure 6B).

### 3.8. Tissue- and Cell-Type-Specific Expression Landscape of FADS1

**Figure 7:**
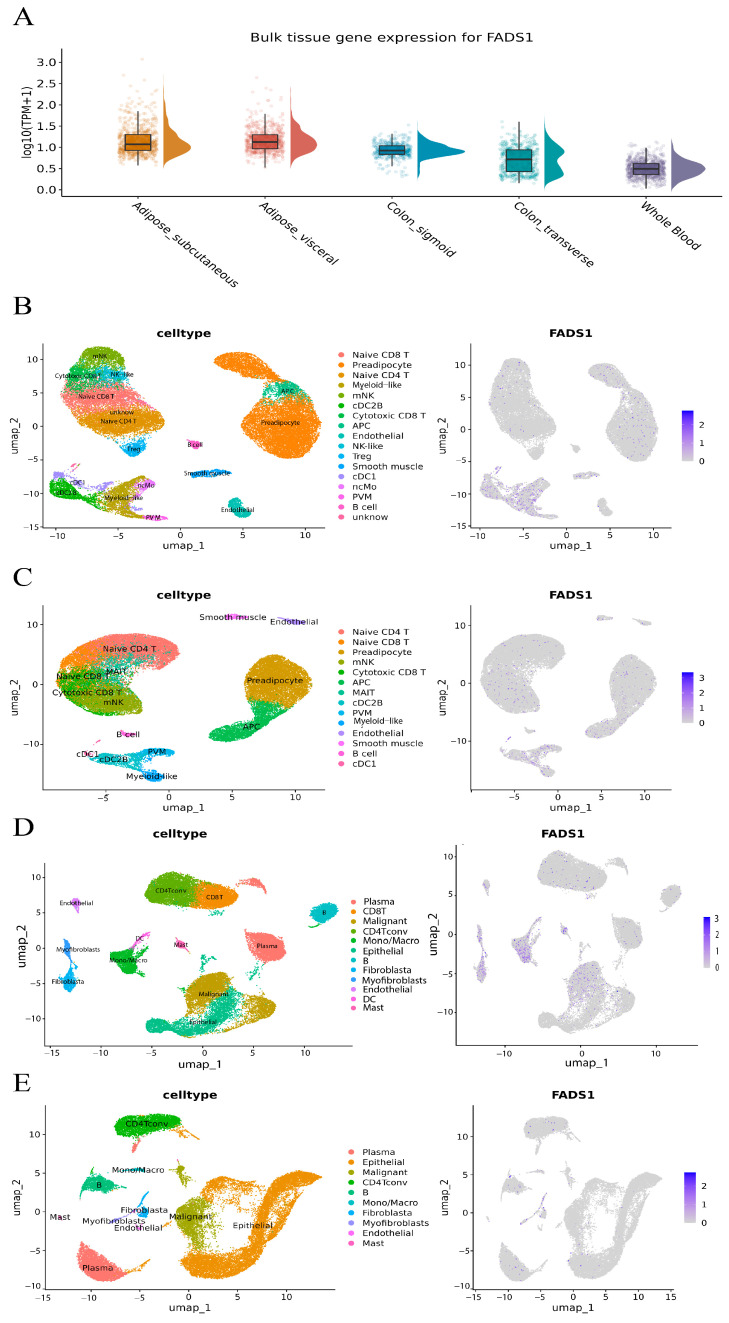
Tissue-and cell-type-specific expression landscape of *FADS1*. (**A**). Bulk RNA-seq-based expression of *FADS1* across GTEx tissues: subcutaneous adipose, visceral adipose, transverse colon, sigmoid colon, and whole blood. Violin and boxplots show log_10_ (TPM + 1)-transformed expression levels per sample; boxes represent interquartile ranges, and density wings indicate distribution; (**B**). Cell type annotation (left) and *FADS1* expression UMAP (right) in adipose tissue of obese individuals. (**C**). Cell type annotation (left) and *FADS1* expression UMAP (right) in adipose tissue of lean individuals. (**D**). Cell type annotation (left) and *FADS1* expression UMAP (right) in CRC tumor tissue. (**E**). Cell type annotation (left) and *FADS1* expression UMAP (right) in adjacent normal colorectal tissue.

To further investigate the potential molecular mechanisms by which the key mediating metabolite may influence CRC development, we focused on the gene with the most functional evidence. Based on the Open Targets Genetics database, *FADS1* and *FADS2* were identified as the top candidates with the highest V2G scores near *rs174546* (0.374 and 0.380, respectively). Given the results of the differential expression and survival analyses, we centered our downstream analysis on *FADS1*, which appeared to play a critical role in CRC pathogenesis and progression. The expression levels of *FADS1* in a variety of tissues in the normal population were assessed using the GTEx database, and *FADS1* was found to be significantly upregulated in both adipose and colonic tissues. Considering that subcutaneous and visceral adipose tissues are key target organs in MetS, and that the transverse colon and sigmoid colon are primary sites of CRC onset, we downloaded bulk RNA-seq data for these four tissues and whole blood and visualized expression using violin plots (Figure 7A). A two-sided Wilcoxon rank-sum test showed that the expression levels of *FADS1* were significantly higher in subcutaneous adipose, visceral adipose, transverse colon, and sigmoid colon tissues compared to whole blood (*p* < 0.001), suggesting local expression of *FADS1* in these tissues.

Given that the *FADS1* gene is significantly overexpressed in adipose tissue and that the transverse and sigmoid colon are key sites for CRC, we further explored its cell type-specific expression patterns using single-cell RNA sequencing data from human white adipose tissue and CRC tumor samples.

In the GSE155960 cohort, single-cell RNA-seq data from obese individuals (*n* = 37,611 cells) and lean individuals (*n* = 40,801 cells) were reanalyzed. Initial clustering identified 21 subpopulations in obese and 18 in lean samples (Figure S13A,B). Based on integrated cell-type markers, 17 cell populations were annotated in the former and 14 in the latter. As shown in Figure 7B,C and Table S27, *FADS1* expression patterns were markedly different between groups: in obese adipose tissue, *FADS1* was significantly enriched in myeloid-like cells, conventional dendritic cells type 1 (cDC1), and conventional dendritic cell type 2 subtype B (cDC2B, permutation test *p* < 0.05). These three types of myeloid immune cells play a key role in adipose tissue homeostasis and metabolic regulation, with cDC1 participating in immune activation through antigen presentation [48], cDC2B having pro-inflammatory properties and driving local inflammatory responses [49], and Myeloid-like cells remodeling the microenvironment, possibly through immune-suppressive functions, which collectively form the core of the obesity-associated chronic inflammation cell population. Whereas fatty acid desaturase 1 encoded by *FADS1* is a key rate-limiting enzyme for the synthesis of polyunsaturated fatty acids (e.g., arachidonic acid), its catalytically generated metabolites (arachidonic acid, leukotrienes, etc.) are known to be potent pro-inflammatory mediators, which have been closely associated with chronic inflammation and the tumor microenvironment [50]. This suggests that inflammation may be one of the mechanisms by which *FADS1* contributes to CRC.

In the GSE166555 cohort, we reanalyzed single-cell RNA-seq data from CRC tumor tissues (*n* = 32,294 cells) and adjacent normal tissues (*n* = 27,247 cells), identifying 22 and 20 subclusters, respectively (Figure S13C,D). Based on integrated cell-type markers, 12 cell populations were annotated in the former and 10 in the latter. As shown in Figure 7D,E and Table S26, cell-type-specific expression of *FADS1* differed significantly between the tumor and adjacent tissues. In tumors, *FADS1*-positive cells were significantly enriched in fibroblasts, myofibroblasts, endothelial cells, dendritic cells (DCs), and malignant (permutation test *p* < 0.05). In contrast, only fibroblasts and myofibroblasts showed detectable *FADS1* expression in normal tissues, suggesting that these cell populations may be responsible for local *FADS1* production in the tumor microenvironment.

Notably, all of these tumor-specific cell types with high *FADS1* expression have previously been reported to be associated with CRC progression: endothelial cells directly accelerate the tumor blood supply, cancer cell extravasation and immune escape through aberrant angiogenesis, disruption of the vascular barrier, and the expression of immunosuppressive molecules (e.g., PD-L1) [51,52]; the abnormal function of DCs, as key regulators of the immune response, can lead to the dysregulation of anti-tumor immunity [53]; the high expression of *FADS1* in malignant epithelial cells themselves may directly activate the pro-cancer metabolic pathway. Taken together, these findings suggest that *FADS1* may serve as an important multicellular co-regulatory node in the CRC microenvironment.

Although current data support the differential and cell-type-specific expression of *FADS1* in CRC-relevant tissues, further experimental validation is required to elucidate the exact mechanisms by which *FADS1* influences tumor development and progression.

### 3.9. PPI Network, Enrichment Analysis and Druggability Assessment

**Figure 8:**
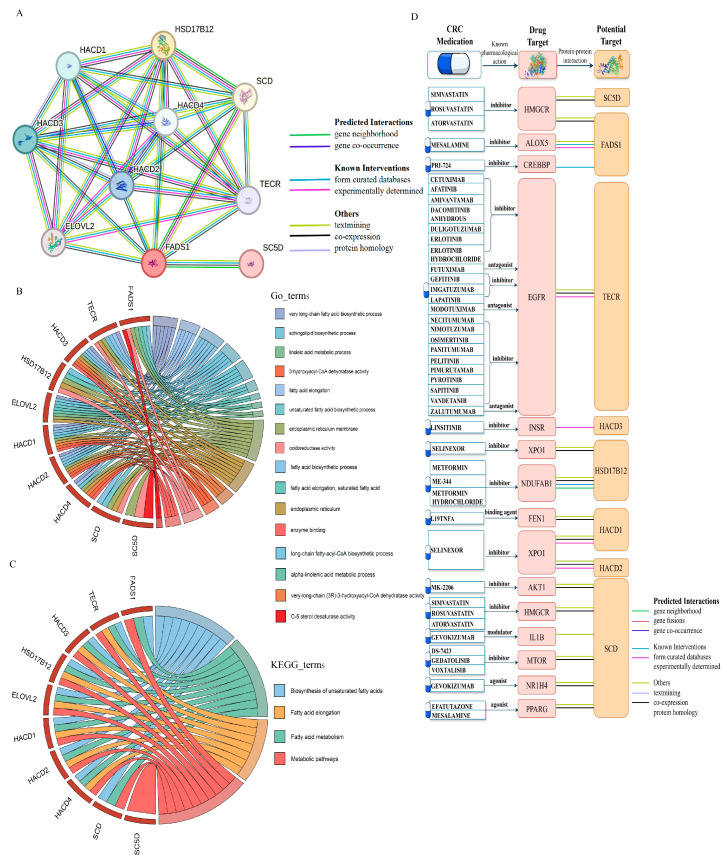
PPI network, functional enrichment and drug target associations of *FADS1*-interacting proteins. (**A**). PPI network of *FADS1* and its nine high-confidence interacting proteins (interaction score ≥ 0.9) using the STRING database. Edge colors indicate different types of evidence supporting each interaction, including predicted interaction (gene neighborhood, gene fusions, co-occurrence), known interaction (experimentally determined, curated database), text mining, co-expression, and protein homology. (**B**). Chord diagrams of enriched GO biological process terms for *FADS1* intercalating proteins. (**C**). Chord diagrams of enriched KEGG pathways for *FADS1* intercalating proteins. In (**B**,**C**), the right semicircles represent significantly enriched GO terms or KEGG pathways, while the left semicircles represent the corresponding proteins involved. (**D**). Interaction landscape between *FADS1*-interacting proteins and known CRC drug targets. The protein structures of drug targets and potential targets were obtained from the SWISS-MODEL website [56,57,58].

We constructed a PPI network for *FADS1* using the STRING database to investigate its interacting partners and their connections to approved drug targets for CRC. The minimum interaction score was set to 0.9, identifying nine high-confidence interacting proteins: *TECR*, *HACD3*, *HSD17B12*, *ELOVL2*, *HACD1*, *HACD2*, *HACD4*, *SCD*, and *SC5D*, which are shown in Figure 8A and Table S27. A functional enrichment analysis revealed that these candidate proteins are primarily involved in lipid biosynthesis, unsaturated fatty acid synthesis, and fatty acid metabolism (Figure 8B,C and Table S28).

To assess the therapeutic potential, we searched DrugBank, Open Targets, and the Therapeutic Targets database for existing drugs or supplements targeting the above proteins (Table S29). Among these, drugs targeting *ELOVL2* include alpha-linolenic acid (ALA). Currently, there are two nutritional supplements—ALA (ligand) and eicosapentaenoic acid (EPA, agonist)—that act on the acyl-CoA (8-3)-desaturase, encoded by *FADS1*. EPA has been used to reduce triglyceride levels in hypertriglyceridemic patients and has shown potential benefits in cystic fibrosis and type 2 diabetic nephropathy. A recent meta-analysis reported that higher serum ALA levels were significantly associated with a reduced CRC risk [54], and EPA has been shown to downregulate *FADS1* expression, with evidence of chemopreventive effects in patients with familial adenomatous polyposis [55]. Taken together, these findings suggest that ALA and EPA, as *FADS1*-targeting nutritional agents, may represent promising candidates for anti-cancer therapy.

Furthermore, we found that 8 out of 10 interacting proteins interacted with known CRC drug targets (Figure 8D and Table S30), further supporting the therapeutic relevance of this network and the potential for targeted intervention strategies centered on *FADS1*-associated pathways.

## 4. Discussion

In this study, we systematically revealed the genetic associations and causal mechanisms between 10 metabolic disease-related phenotypes and CRC by integrating MR analyses with large-scale population genetic data. Through genetic correlation analysis, we found significant genetic correlations between obesity-related indicators (BMI, WC), BMR, diabetes-related indicators (IR), and MetS with CRC risk, a result consistent with previous observational studies [3,4]. The subsequent two-sample MR analysis further confirmed the causal nature of these associations, aligning with prior MR findings on obesity, MetS, and CRC risk [5,6]. Building on these results, we conducted a comprehensive investigation of plasma metabolites to search for common mediators in the metabolic disease–to–CRC pathway. A two-step MR analysis revealed that mannonate, an exogenous metabolite, exhibited high mediation proportions in the pathways from four metabolic traits to CRC (BMI: 65.49%, WC: 50.61%, BMR: 63.08%, and MetS: 32.52%), suggesting a potential hub role in the metabolic-oncologic transition and its promise as an early biomarker. In addition, several other metabolites—1-linolenoyl-2-linolenoyl-GPC (18:2/18:3), the mannose–to–trans-4-hydroxyproline ratio, and plasma free asparagine—were found to significantly mediate the causal effects of obesity-related traits and MetS on CRC. Notably, our findings for 1-linolenoyl-2-linolenoyl-GPC (18:2/18:3) are consistent with previous MR studies, indicating that lower circulating levels of this metabolite are associated with increased CRC risk [59]. In our analysis, 1-linolenoyl-2-linolenoyl-GPC (18:2/18:3) mediated up to 20.44% and the causal SNP (*rs174546*) of this metabolite with CRC was significantly enriched in several pathways related to fatty acid metabolism, especially linoleic acid metabolism, α-linolenic acid (omega 3) and linoleic acid (omega 6) acid metabolism. A large population-based cohort study has similarly found that the metabolic pathways with the most significant differences in plasma metabolites in CRC patients compared to healthy populations are α-linolenic and linoleic acid metabolism [60]. Gene annotation and TCGA-based differential expression analyses further revealed significant upregulation of the *FADS1/2/3* gene cluster—located near *rs174546*—in CRC tissues, with high *FADS1* expression correlating with poor prognosis. These findings suggest that this gene may drive CRC progression by regulating phosphatidylcholine synthesis and fatty acid metabolism. Previous studies have shown that *FADS1* genetic variants alter the balance of arachidonic acid (AA) and other long-chain polyunsaturated fatty acid derivatives, modulating inflammation in adipose tissue triggered by dietary linoleic acid (LA). *FADS1* has also been directly implicated in CRC pathogenesis. A recent study demonstrated that the *FADS1*-AA axis promotes prostaglandin E_2_ (PGE2) production in CRC cells and reshapes the gut microbiota, thereby directly contributing to tumor development [50]. Under obesity conditions, pro-inflammatory DCs and immunosuppressive myeloid cells in adipose tissue and the tumor microenvironment may synergistically reprogram the immune landscape toward a tumor-promoting state. Therefore, *FADS1*-driven immune-inflammatory reprogramming in adipose tissue may be a potential mechanism linking obesity with elevated CRC risk. Although our two-step MR and colocalization results implicate a potential role for genes near the *rs174546* locus and their associated fatty acid metabolic pathways in mediating the relationship between metabolic phenotypes and CRC risk, these inferences remain grounded in aggregated genetic evidence rather than direct functional experimentation. Future studies should therefore validate these candidates and elucidate causal mechanisms using in vitro cellular models, animal models, and intervention trials. In addition, investigations using matched multi-omics datasets from the same individuals (e.g., paired transcriptomic and metabolomic profiles) will be critical for clarifying the potential biomarker roles and molecular mechanisms of *FEN1*, *FADS1*, *FADS3*, *RAB3IL1* and *NRBP1* in CRC.

In summary, our study implicates several shared plasma metabolites and fatty-acid metabolic pathways in the progression from metabolic diseases to CRC, with pathways related to linoleic acid and α-linolenic acid metabolism being particularly prominent. Genetically anchored metabolomic signals provide new insights into how metabolic disorders may contribute to CRC and nominate a set of candidate metabolites that warrant further evaluation as potential biomarkers for early detection or risk stratification. From a translational perspective, two potential avenues emerge. First, metabolites that are analytically validated and suitable for high-throughput measurement (e.g., 1-linolenoyl-2-linolenoyl-GPC (18:2/18:3)) could be incorporated into existing risk prediction models. Second, if certain metabolites show strong correlation with *FADS1* expression, then nutritional or pharmacological interventions targeting *FADS1* or related metabolic pathways—such as the phosphatidylcholine/phosphatidylethanolamine biosynthetic pathway (involving 1-linolenoyl-2-linolenoyl-GPC (18:2/18:3)) and broader fatty-acid metabolic pathways—may represent promising strategies to prevent or mitigate the risk of metabolically driven cancers. It must be emphasized that several critical steps remain between statistical association and clinical application. Firstly, analytical validation is required [61]; secondly, epidemiological validation must be conducted in independent and distinct cohort populations to assess generalizability and predictive improvement [62]; finally, in vivo and in vitro experiments and early intervention studies are necessary to establish actionability and validate mechanisms [63].

This study has several advantages: (1) it is the first comprehensive and systematic identification of genetic associations between metabolic diseases and CRC, as well as the shared plasma metabolites and related genes involved in their causal pathways, revealing key metabolic hubs such as mannonate, 1-linolenoyl-2-linolenoyl-GPC (18:2/18:3) and others, which can help to provide new directions for metabolic disease patients to reduce the risk of CRC; (2) its use of bidirectional MR and two-step MR enabled the construction of triangular causal chains, effectively minimizing confounding and reverse causality bias; and (3) it integrated multi-omics datasets, including GWAS data, transcriptome, survival outcomes, single-cell RNA sequencing, and drug target information to validate the biological consistency of the metabolite–gene pathway.

Despite employing multiple genetic epidemiological approaches to strengthen causal inference, this study has several limitations. (1) In the two-sample MR analyses linking metabolic disease-related phenotypes and plasma metabolites, although FDR correction and multiple sensitivity tests were applied, residual multiple-testing bias and false-positive risks remain a concern, given the large-scale evaluation of 1400 metabolites. Replication of the key findings in independent metabolomics cohorts is therefore warranted. (2) In MR analyses assessing shared metabolites’ roles in CRC, a nominal significance threshold of *p* < 0.05 was used. Although the inclusion of BWMR and additional sensitivity analyses supported robustness, the possibility of false positives cannot be fully excluded. (3) Several putative mediating metabolites (e.g., phosphate–to–mannose ratio, plasma free asparagine) did not reach statistical significance in the replication dataset, despite showing consistent effect directions, highlighting the need for larger sample sizes to validate these associations. (4) All the data analyzed were derived from populations of European ancestry. Results may thus be influenced by ancestry-specific linkage disequilibrium structures, limiting their generalizability to other populations, and reflecting the reliance on publicly available datasets. Future studies should include more diverse populations to enhance the generalizability and external validity of the findings. (5) Finally, while summary-level MR and colocalization provide strong genetic evidence consistent with causality, they cannot by themselves establish molecular mechanisms or substitute for direct functional validation (e.g., in vitro, in vivo, or interventional studies) to confirm that the implicated metabolites or genes causally drive CRC development.

## 5. Conclusions

We leveraged human genomics and plasma metabolomics to map causal pathways from metabolic disorders to CRC risk and to pinpoint shared metabolite mediators. Our analyses reveal that mannonate centrally mediates the effects of obesity, BMR, and MetS on CRC susceptibility. Additionally, the lipid metabolite 1-linolenoyl-2-linolenoyl-GPC (18:2/18:3) jointly mediates the impacts of BMI, WC, and MetS on CRC risk. Finally, *FADS1* emerged as a pivotal determinant linking obesity, MetS, and elevated CRC risk. These findings provide novel insights into the shared biological mechanisms underlying the causal link between metabolic disorders and increased CRC susceptibility.

## Figures and Tables

**Table 1:** Results of LDSC analysis of metabolism-related phenotypes and colorectal cancer.

Metabolism-Related Phenotypes	Single Trait			Cross Trait		
	h^2^	se	*p*	h^2^	se	*p*
BMI	0.247	0.008	<0.001	0.126	0.043	0.003
WC	0.195	0.007	<0.001	0.156	0.040	<0.001
BMR	0.305	0.012	<0.001	0.108	0.039	0.005
HTN	0.885	0.037	<0.001	0.016	0.056	0.766
HLD	0.011	0.003	<0.001	0.014	0.107	0.900
T2DM	0.035	0.003	<0.001	0.088	0.062	0.158
IR	0.057	0.012	<0.001	0.473	0.142	<0.001
Gout	0.002	0.001	0.088	0.049	0.235	0.835
MetS	0.128	0.005	<0.001	0.197	0.044	<0.001
NAFLD	0.008	0.001	<0.001	0.056	0.119	0.638
CRC	0.099	0.021	<0.001			

h^2^, heritability; se, standard error; *p*, *p*-value; rg, genetic correlation.

**Table 2:** Results of multiple linear regression.

Variable	Beta	SE	95% CI	*p*-Value
PC ~ BMI/WC + age + sex + recruitment center (center) + metabolite measurement batch + sample processing delay time				
BMI (adjusted)	−0.020	0.000	(−0.021, −0.019)	<0.001
WC (adjusted)	−0.008	0.000	(−0.008, −0.007)	<0.001
PC ~ BMI + WC + age + sex + recruitment center (center) + metabolite measurement batch + sample processing delay time				
BMI (adjusted)	−0.015	0.001	(−0.016, −0.013)	<0.001
WC (adjusted)	−0.003	0.000	(−0.003, −0.002)	<0.001

**Table 3:** Results of multivariate Cox proportional risk regression.

Variable	HR	SE	95% CI	*p*-Value
PC (adjusted)	1.207	0.026	(1.147, 1.270)	<0.001
PC-male (adjusted)	1.341	0.036	(1.250, 1.438)	<0.001
PC-female (adjusted)	1.046	0.038	(0.971, 1.127)	0.233

PC-male (adjusted) and PC-female (adjusted) are the results of stratified analysis by gender and adjusted for covariates, respectively.

## Data Availability

All the data used in this study were obtained from publicly available sources. The sources of all GWAS data are indicated in the “Data sources” section of the manuscript. The UK Biobank data used in this study are available by submitting a research application (application portal: https://www.ukbiobank.ac.uk/enable-your-research/register, accessed on 1 April 2025); the current study was conducted under application ID 94184. SNP functional annotation was performed using the SNPnexus platform (https://www.snp-nexus.org, accessed on 6 December 2024). RNA-seq data for CRC patients were obtained from TCGA project (https://portal.gdc.cancer.gov/, accessed on 17 December 2024) and transcriptome expression data for normal tissues were obtained from the GTEx project (https://gtexportal.org/, accessed on 17 December 2024). For the tissue- and cell-type-specific analysis of gene expression, we used single-cell RNA-seq data from the GSE166555 cohort in the TISCH database (http://tisch.comp-genomics.org/, accessed on 23 December 2024) and the GSE155960 cohort in the GEO database (https://www.ncbi.nlm.nih.gov/geo/, accessed on 23 December 2024). The PPI analysis was performed using the STRING database (https://cn.string-db.org/, accessed on 30 December 2024), and functional annotation and pathway enrichment were conducted using the DAVID database (https://davidbioinformatics.nih.gov/, accessed on 30 December 2024). Drug-target information was retrieved from the DrugBank (https://go.drugbank.com, accessed on 30 December 2024), Open Targets (https://platform.opentargets.org/, accessed on 30 December 2024), and the Therapeutic Target Database (https://db.idrblab.net/ttd/, accessed on 30 December 2024). When using the above data sources, researchers should follow the usage guidelines of each database and cite the original published literature. All the original data used in this study are available through the links provided in the text.

## Supplementary Materials

The following supporting information can be downloaded at https://www.mdpi.com/article/10.3390/biomedicines13102433/s1, Notes in Supplementary Information Note S1–S4: Supplementary Note S1: Instrumental variable selection and quality-control procedures; Supplementary Note S2: The criteria for selecting the final causal inference method; Supplementary Note S3: Colocalization analysis and threshold settings; Supplementary Note S4: Association between BMI and WC and plasma PC levels in UK Biobank; Tables in Supplementary Tables S1–S30: Table S1. Information on data sources for exposure, mediation and outcome [64,65]; Table S2. Instrumental variables used in MR analysis of the association between metabolic related phenotypes; Table S3. Genetic instruments for causal inference of BMI and colorectal cancer; Table S4. Genetic instruments for causal inference of WC and colorectal cancer; Table S5. Genetic instruments for causal inference of BMR and colorectal cancer; Table S6. Genetic instruments for causal inference of IR and colorectal cancer; Table S7. Genetic instruments for causal inference of MetS and colorectal cancer; Table S8. Post hoc power calculations for the selected SNPs; Table S9. Tests of pleiotropy and heterogeneity of SNPs for metabolic phenotypes causatively associated with colorectal cancer; Table S10. Statistical power for MR Analysis of SNPs was selected; Table S11. Full result of MR estimates for the association between the five metabolism-related phenotypes and plasma metabolites by the primary analysis method; Table S12. Full result of MR estimates for the association between the 5 metabolism-related phenotypes and plasma metabolites by BWMR; Table S13. MR findings for metabolites commonly associated with the 4 metabolic phenotypes; Table S14. Tests of pleiotropy and heterogeneity of plasma metabolite SNPs; Table S15. Instrumental variables for plasma metabolites used in MR analyses of associations between metabolic disease-related plasma metabolites and colorectal cancer; Table S16. MR findings on metabolites co-associated with metabolic phenotypes and colorectal cancer; Table S17. Statistical power for MR Analysis of SNPs was selected; Table S18. Reverse validation of MR results; Table S19. Results of 2-step MR analysis in the colorectal cancer validation dataset; Table S20. Tests of pleiotropy and heterogeneity of SNPs used in 2-step MR analysis in the colorectal cancer validation dataset; Table S21. Results of co-localization with colorectal cancer by common mediators in metabolism-related phenotypes and causal pathways of colorectal cancer; Table S22. Results of the two-step mediation analysis; Table S23. Linear and Cox-regression analysis in the UK Biobank; Table S24. Results of pathway analysis at *rs35540647*, *rs174546*, *rs1260326* in SNP-nexus; Table S25. Results of univariate Cox regression; Table S26. Displacement analysis of differential expression of *FADS1* in cells; Table S27. Protein interaction network of *FADS1* protein with its interacting key proteins; Table S28. GO enrichment, KEGG pathway analysis and existing drugs for *FADS1* protein with its interacting key proteins; Table S29. Existing drugs for *FADS1* interacting proteins. Table S30. Protein–protein interaction network between *FADS1* interacting proteins and known CRC drug targets; Figures in Supplementary Figures S1–S13: Figure S1 Conceptual diagram of MR analysis. BMI, body mass index; WC, waist circumference; BMR, basal metabolic rate; MetS, metabolic syndrome; NAFLD, non-alcoholic fatty liver disease; IR, insulin resistance; Figure S2 Scatterplot of effects of metabolic disease-related phenotypes on colorectal cancer; A. Effect of BMI on colorectal cancer. B. Effect of WC on colorectal cancer. C. Effect of BMR on colorectal cancer. D. Effect of IR on colorectal cancer. E. Effect of MetS on colorectal cancer. Figure S3 Leave-One-Out plot of effects of metabolic disease-related phenotypes on colorectal cancer; A. Effect of BMI on colorectal cancer. B. Effect of WC on colorectal cancer. C. Effect of BMR on colorectal cancer. D. Effect of IR on colorectal cancer. E. Effect of MetS on colorectal cancer. Figure S4 Circular heat map of lipid metabolites and metabolic disease-related phenotypes; Figure S5 Circular heat map of amino acid metabolites and metabolic disease-related phenotypes; Figure S6 Circular heat map of unknown metabolites and metabolic disease-related phenotypes; Figure S7 Circular heat map of 6 types of metabolites (xenobiotics, carbohydrate, cofactors and vitamins, peptide, energy, nucleotide and partially characterized molecules) and metabolic disease-related phenotypes; Figure S8 Leave-One-Out plot of effects of intermediate metabolites on colorectal cancer; A. Effect of glucose-to-mannose ratio on colorectal cancer. B. Effect of mannonate levels on colorectal cancer. C. Effect of phosphate to mannose ratio on colorectal cancer. D. Effect of Plasma free asparagine levels on colorectal cancer. E. Effect of mannose to trans-4-hydroxyproline ratio on colorectal cancer. F. Effect of N6-carbamoylthreonyladenosine levels on colorectal cancer. G. Effect of 1-linoleoyl-2-linolenoyl-GPC (18:2/18:3) levels on colorectal cancer. H. Effect of Oleoyl-linoleoyl glycerol (18:1/18:2) levels on colorectal cancer. I. Effect of Adenosine 5’ diphosphate (ADP) to aspartate ratio on colorectal cancer. Figure S9 LocusZoom plots of 1-linoleoyl-2-linolenoyl-GPC (18:2/18:3) levels and colorectal cancer. Figure S10 Mediating ratios of plasma free asparagine levels, mannose–to–trans-4-hydroxyproline ratio and glucose–to–mannose ratio in the causal pathway; Figure S11 Survival curves for genes near *rs174546*. HR and confidence intervals from univariate Cox proportional hazards regression; A. Survival curve of *DAGLA*. B. Survival curve of *FADS1*. C. Survival curve of *FADS2*. D. Survival curve of *FADS3*. E.Survival curve of *FEN1*. F. Survival curve of *MYRF*. G. Survival curve of *RAB3IL1*. Figure S12 Survival curves for genes near *rs1260326*.A. Survival curve of *FNDC4* B. Survival curve of *GCKR*. C. Survival curve of *IFT172*. D. Survival curve of *KRTCAP3*. E.Survival curve of *NRBP1*. F. Survival curve of *PPM1G*. G. Survival curve of *ZNF512*. Figure S13 Single-cell transcriptomic profiling of adipose and colorectal tissues. A. UMAP clustering (left) and dot plot of the top 2 expressed genes (right) in single-cell data from adipose tissue of obese individuals (GSE155960); B. UMAP clustering (left) and dot plot of the top 2 expressed genes (right) in adipose tissue of lean individuals (GSE155960); C. UMAP clustering (left), top 2 expressed genes (upper right), and number of differentially expressed genes per cluster (lower right) in CRC tumor tissue (GSE166555); D. Same analyses as in (C) for adjacent normal colorectal tissue.

## Abbreviations

The following abbreviations are used in this manuscript:

CRC	colorectal cancer
PPI	protein–protein interaction
IARC	international agency for research on cancer
MetS	metabolic syndrome
IR	insulin resistance
BMI	body mass index
T2DM	type 2 diabetes mellitus
NAFLD	non-alcoholic fatty liver disease
MR	Mendelian randomization
IVs	instrumental variables
RCTs	randomized controlled trials
TSMR	two-sample Mendelian randomization
GWAS	genome-wide association study
WC	waist circumference
BMR	basal metabolic rate
HTN	hypertension
HLD	hyperlipidemia
MRC IEU	Medical Research Council Integrative Epidemiology Unit
LDSC	linkage disequilibrium score regression
PC	phosphatidylcholine
UKB	UK biobank
SNP	single-nucleotide polymorphism
TCGA	The Cancer Genome Atlas
LD	linkage disequilibrium
IVW	inverse variance weighted
BWMR	Bayesian weighted Mendelian randomization
FDR	false discovery rate
PPH4	posteriori probability of H4
OS	overall survival
GEO	gene expression omnibus
TISCH	tumor immune single-cell hub
UMAP	uniform manifold approximation and projection
GO	gene ontology
KEGG	Kyoto encyclopedia of genes and genomes
DAVID	Database for Annotation, Visualization and Integrated Discovery
cDC1	conventional dendritic cells type 1
cDC2B	conventional dendritic cell type 2 subtype B
DCs	dendritic cells
ALA	alpha-linolenic acid
EPA	eicosapentaenoic acid
AA	arachidonic acid
LA	linoleic acid
