# Supplementary material for: Shared Plasma Metabolites Mediate Causal Effects of Metabolic Diseases on Colorectal Cancer: A Two-Step Mendelian Randomization Study
Source: Biomedicines. 2025 Oct 6;13(10):2433. doi: 10.3390/biomedicines13102433 (PMC12561098; doi:10.3390/biomedicines13102433)
Supplement: Supplementary file 1 [file biomedicines-13-02433-s001.zip › additional file.pdf]

Shared Plasma Metabolites Mediate Causal Effects of Metabolic Diseases on Colorectal Cancer : A Two-Step Mendelian Randomization Study

Supplementary Information

Table of contents:

Supplementary Note S1: Instrumental variable selection and quality-control procedures

Supplementary Note S2: The criteria for selecting the final causal inference method

Supplementary Note S3: Colocalization analysis and threshold settings

Supplementary Note S4: Association between BMI and WC and plasma PC levels in UK Biobank

Supplementary Note S1: Instrumental variable selection and quality-control procedures

To screen for genetic variants suitable for MR analysis, the following criteria were used in this study:(1) Initially, 5.0×10^{-8} was used as the threshold of genetic significance for extracting IVs; however, as only a few SNPs for plasma metabolites met this criterion, to further explore the potential mediating role of plasma metabolites between metabolic diseases and CRC, the subsequent adjustment of the threshold to 5.0×10^{-6} [1]; (2) calculate linkage disequilibrium (LD) between SNPs using the European 1000 Genomes Project sample data, and screen out SNPs with an LD correlation coefficient (r^2) of 0.001 in a 10000 kb cluster window, and only variants with the lowest *P*-value were retained; (3) SNPs with minor allele frequency (MAF) ≤ 0.01 were excluded to ensure robust results; (4) During the harmonization of exposure and outcome datasets, non-palindromic SNPs with ambiguous allele frequencies (i.e., A/T or G/C SNPs) were removed; and (5) Potential associations between the selected SNPs and known confounders—including alcohol consumption and smoking—were assessed using the "*LDlinkR*" (v1.4.0) R package, and any SNPs associated with these confounders were excluded from further analysis [2].

For IVs that met 3 core assumptions of MR, we implemented a series of rigorous quality control processes to ensure the reliability of the IVs. First, the "*RadialMR*" v1.1 R package was used to detect heterogeneity and filter out anomalous pleiotropic SNPs [3]. Specifically, we applied Cochran's Q-test and Rucker's Q-test to assess the horizontal and directional pleiotropy, respectively, with the parameters set where the parameters were set to a significance level of $\alpha = 0.05$, a

weighting factor of 1, a convergence tolerance threshold of 0.0001 (based on the inverse variance weighted radial model, IVW-radial), and a significance level α = 0.05 and a weighting coefficient of 1 (based on the Egger radial regression model, Egger-radial). At a *P*-value threshold of 0.05, identified anomalous SNPs were excluded. Subsequently, we calculated the *F*-statistic to measure the potency of the IVs, which was calculated as $F = \frac{R^2\,(N-2)}{1-R^2}$, where *N* represents the exposed sample size, and R^2 denotes the proportion of phenotypic variance explained by genetic variance. R^2 Calculated by effect size (*β*) and standard error (*SE*), the formula is

$$R^2 = \frac{2 \times \beta^2 \times EAF \times (1 - EAF)}{2 \times \beta^2 \times EAF \times (1 - EAF) + [2 \times SE^2 \times N \times EAF \times (1 - EAF)}$$

calculated from [4,5]. To minimize bias from weak instruments, only SNPs with an *F*-statistic ≥ 10 were retained.

Supplementary Note S2: The criteria for selecting the final causal inference method

(1) If multiple tests (Cochran's Q, MR-Egger intercept, and MR-PRESSO) showed no significant bias (*P* > 0.05), IVW was selected as the primary estimator due to its optimal statistical power in the absence of pleiotropy;

(2) if directional pleiotropy was present (MR-Egger intercept *P* ≤ 0.05) but the Rucker's Q test supports model homogeneity (*P* > 0.05), the IVW assumptions were violated, and MR-Egger was selected as the primary method;

(3) If both directional pleiotropy and heterogeneity were present (Rucker's Q *P* ≤ 0.05), weighted models were adopted to estimate causal effects.

Supplementary Note S3: Colocalization analysis and threshold settings

In this study, we used the "*coloc*" v5.2.3 R package to assess colocalization by calculating posterior probabilities in a Bayesian framework under 5 hypotheses: H_0 (no association with either trait in the region), H_1 (only associated with CRC), H_2 (only associated with metabolite levels), H_3 (both traits were associated through different causal variants), and H_4 (both traits shared the same causal variant). Default priori probabilities were used for the study: p_1 (a priori probability that the exposure has a causal variant) = 1.0×10^{-4}, p_2 (a priori probability that the outcome has a causal variant) = 1.0×10^{-4}, and p_{12} (a priori probability that the exposure and outcome share the same causal variant) = 1.0×10^{-5}. To estimate the a posteriori probability of H_4 (PPH_4) in the genomic region where each metabolite SNP-IV is located, the study analyzed common variants (MAF > 0.01) within 500kb upstream and downstream of the SNPs [6]. When PPH_4 exceeded 0.70, the metabolite in this region was considered to have colocalized features with CRC, thus supporting the hypothesis of a shared pathogenic SNP between 2 traits [7].

Supplementary Note S4: Association between BMI and WC and plasma PC levels in UK Biobank

Method: In this study, multiple linear regression analyses were used to assess the association between baseline BMI, WC and plasma PC levels in 265,603 individuals from UKB. Participants from UKB whose plasma metabolite measurements were obtained from a high-throughput NMR-based metabolic biomarker analysis platform developed by Nightingale Health Ltd. were included.

Participants who already had CRC at baseline were excluded to account for confounding factors. For covariates, we included age at recruitment (data field: 21022), sex (data field: 31), recruitment center (data field: 54), metabolite measurement batch (data field: 20282) and metabolite processing delay time (data field: 23658). Next, BMI (data field: 21001), and WC (data field: 48) were used as the independent variables, and plasma PC level as the dependent variable, respectively, which were subjected to a rank-based normalization using the RankNorm function in the "*RNOmni*" v1.0.1.2 R package. If the significance *P*-value of the regression coefficients for BMI and WC reaches $P < 0.05$, the results are considered significant, indicating that there is a significant correlation between baseline BMI, WC, and plasma PC levels.

To further assess whether baseline plasma PC levels were associated with the risk of CRC, this study conducted multivariable Cox proportional hazards regression analysis, adjusting for the same covariates (age, sex, recruitment center, metabolite measurement batch, and delayed time to metabolite processing). Incident CRC cases were defined as: (1) diagnosis codes C18 (colon cancer), C19 (sigmoid colon cancer), or C20 (rectal cancer) based on ICD-10; (2) surgical records of transcolorectal resections (OPCS-4 codes, field 20004, or operative records); and (3) CRC-related death records. Participants who already had CRC at baseline and cases in which the event date was not recorded were excluded. Time to event was calculated as "date of onset (or date of last follow-up) minus date of registration", with a focus on events occurring within 15 years of enrolment. Cox regression analyses were performed

using the *coxph()* function in the R package "*Survival*" v.3.7-0. If the significance P-value of the regression coefficient of the PC reaches $P < 0.05$, the result is considered significant, indicating a significant association between baseline plasma PC levels and CRC risk.

References

[1] Wang Q, Shi Q, Wang Z, Lu J, Hou J. Integrating plasma proteomes with genome-wide association data for causal protein identification in multiple myeloma. BMC Med 2023;21:377. https://doi.org/10.1186/s12916-023-03086-0.

[2] Myers TA, Chanock SJ, Machiela MJ. LDlinkR: An R Package for Rapidly Calculating Linkage Disequilibrium Statistics in Diverse Populations. Front Genet 2020;11:157. https://doi.org/10.3389/fgene.2020.00157.

[3] Bowden J, Spiller W, Del Greco M F, Sheehan N, Thompson J, Minelli C, et al. Improving the visualization, interpretation and analysis of two-sample summary data Mendelian randomization via the Radial plot and Radial regression. Int J Epidemiol. 2018 ;47(6):2100. doi: 10.1093/ije/dyy265.

[4] Gill D, Efstathiadou A, Cawood K, Tzoulaki I, Dehghan A. Education protects against coronary heart disease and stroke independently of cognitive function: evidence from Mendelian randomization. Int J Epidemiol 2019;48:1468–77. https://doi.org/10.1093/ije/dyz200.

[5] Palmer TM, Lawlor DA, Harbord RM, Sheehan NA, Tobias JH, Timpson NJ, et al. Using multiple genetic variants as instrumental variables for modifiable risk factors. Stat Methods Med Res 2012;21:223–42. https://doi.org/10.1177/0962280210394459.

[6] Yazdanpanah M, Yazdanpanah N, Gamache I, Ong K, Perry JRB, Manousaki D. Metabolome-wide Mendelian randomization for age at menarche and age at natural menopause. Genome Med 2024;16:69. https://doi.org/10.1186/s13073-024-01322-7.

[7] Zheng S, Tsao PS, Pan C. Abdominal aortic aneurysm and cardiometabolic traits share strong genetic susceptibility to lipid metabolism and inflammation. Nat Commun 2024;15:5652. https://doi.org/10.1038/s41467-024-49921-7.
