# Supplementary material for: Shared Plasma Metabolites Mediate Causal Effects of Metabolic Diseases on Colorectal Cancer: A Two-Step Mendelian Randomization Study
Source: Biomedicines. 2025 Oct 6;13(10):2433. doi: 10.3390/biomedicines13102433 (PMC12561098; doi:10.3390/biomedicines13102433)
Supplement: Supplementary file 1 [file biomedicines-13-02433-s001.zip › Supplementary Figure S1-S13.pdf]

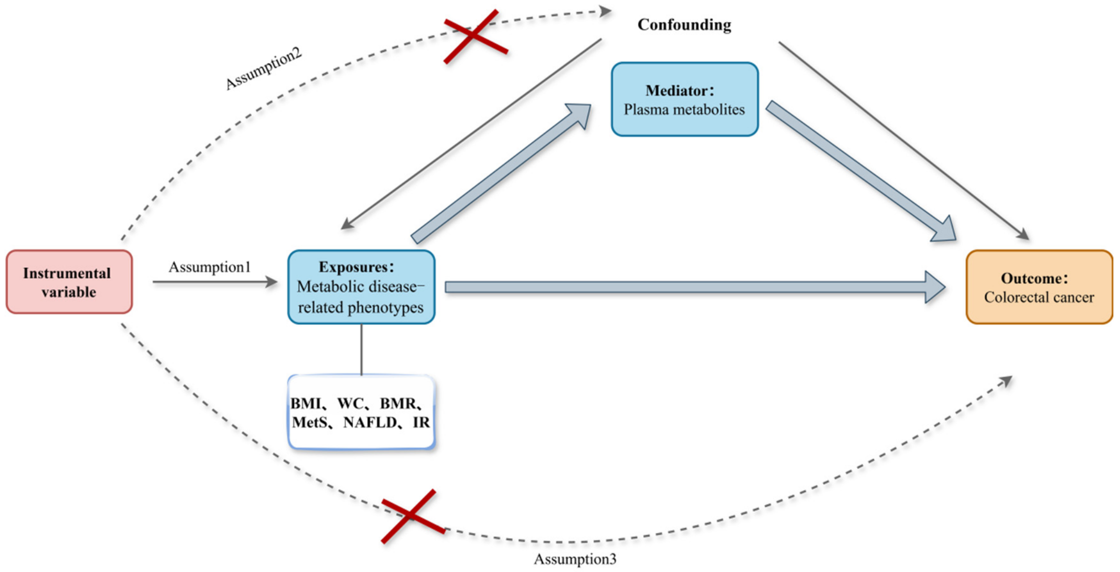

Figure S1. Conceptual diagram of MR analysis. BMI, body mass index; WC, waist circumference; BMR,basal metabolic rate; MetS,metabolic syndrome; NAFLD,non-alcoholic fatty liver disease; IR,insulin resistance

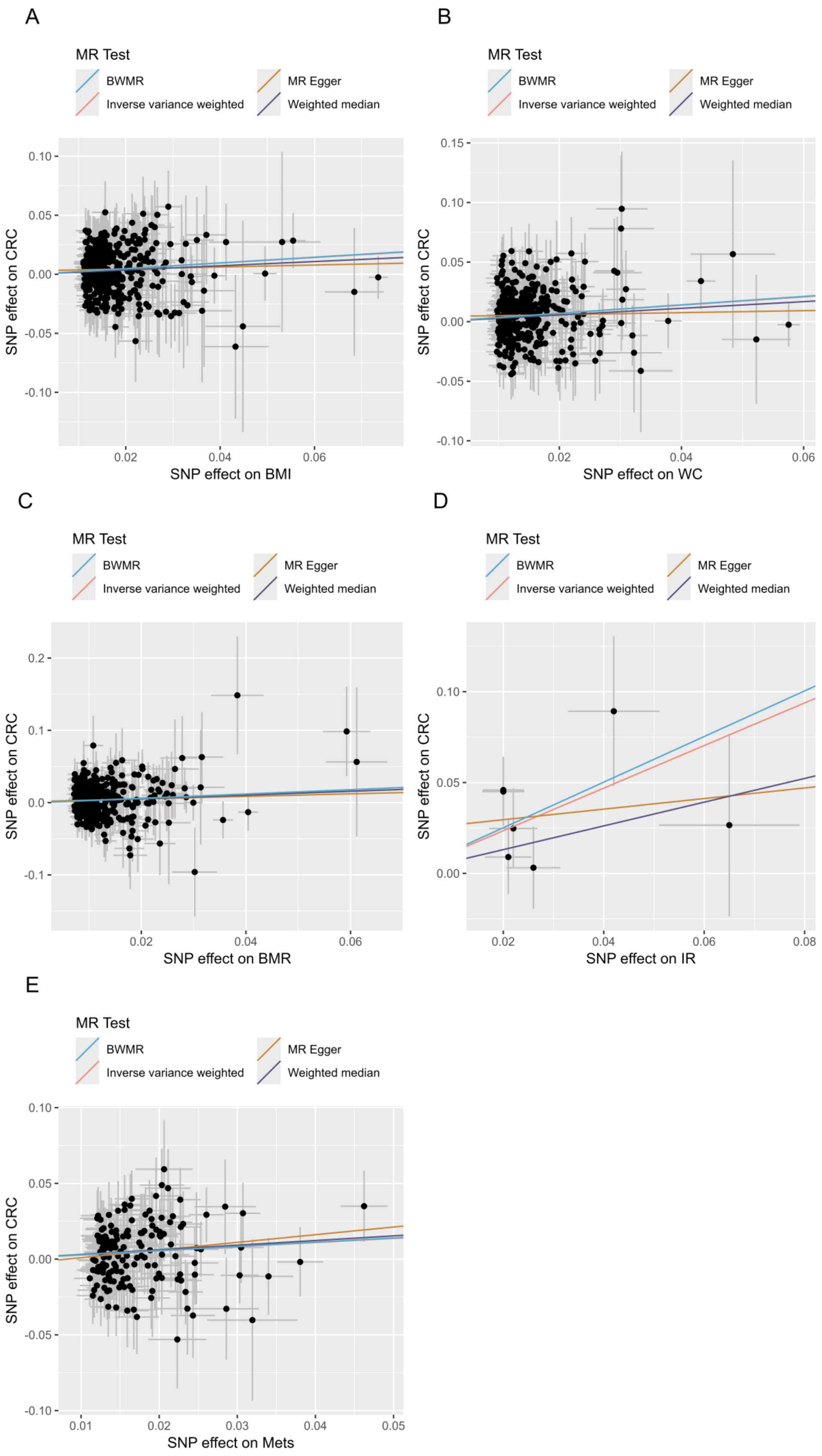

Figure S2. Scatterplot of effects of metabolic disease-related phenotypes on colorectal cancer. (A). Effect of BMI on colorectal cancer. **(B)**. Effect of WC on colorectal cancer. **(C)**. Effect of BMR on colorectal cancer. **(D)**. Effect of IR on colorectal cancer. **(E)**. Effect of MetS on colorectal cancer.

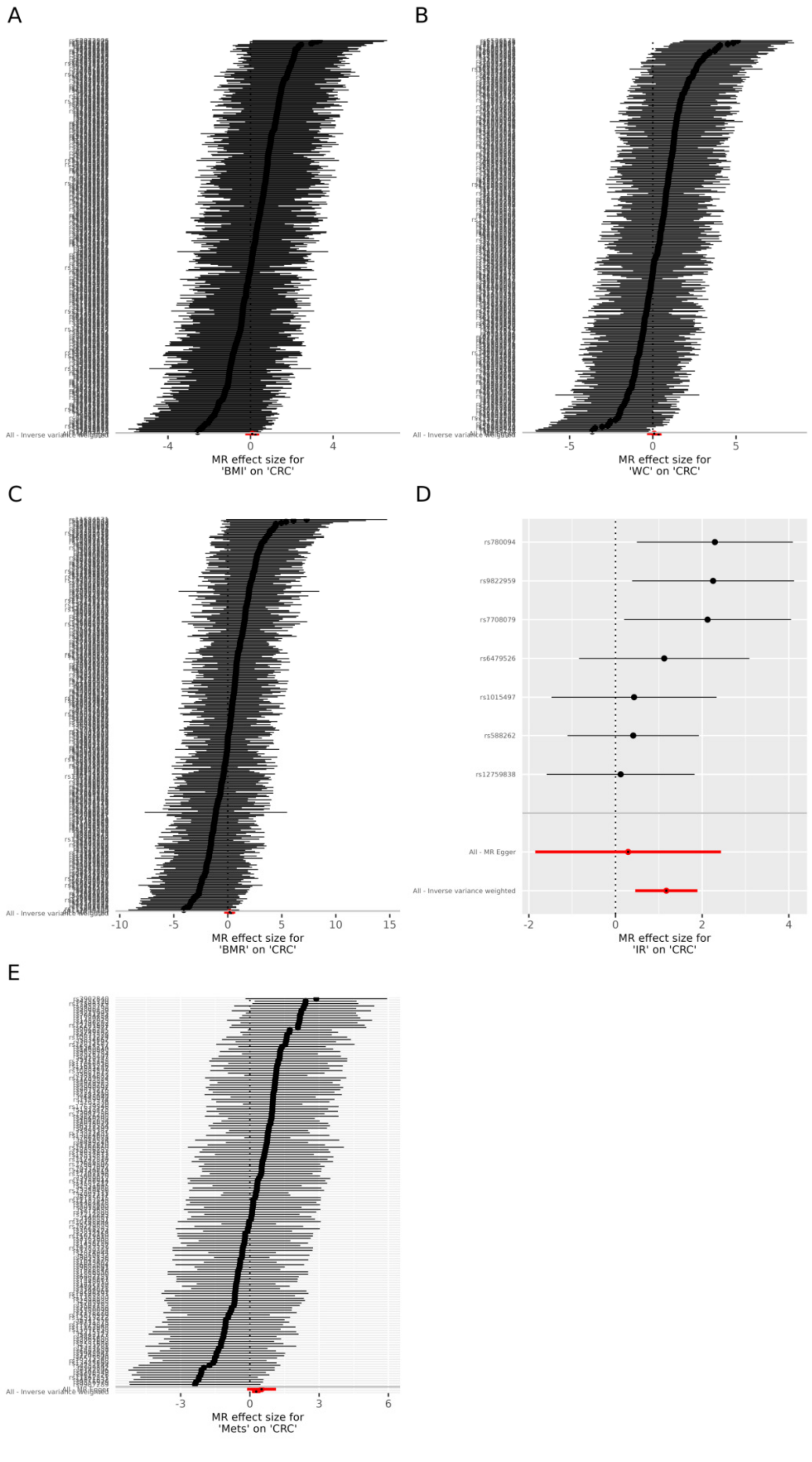

Figure S3. Leave-One-Out plot of effects of metabolic disease-related phenotypes on colorectal cancer. (A). Effect of BMI on colorectal cancer. **(B)**. Effect of WC on colorectal cancer. **(C)**. Effect of BMR on colorectal

cancer. **(D)**. Effect of IR on colorectal cancer. **(E)**. Effect of MetS on colorectal cancer.

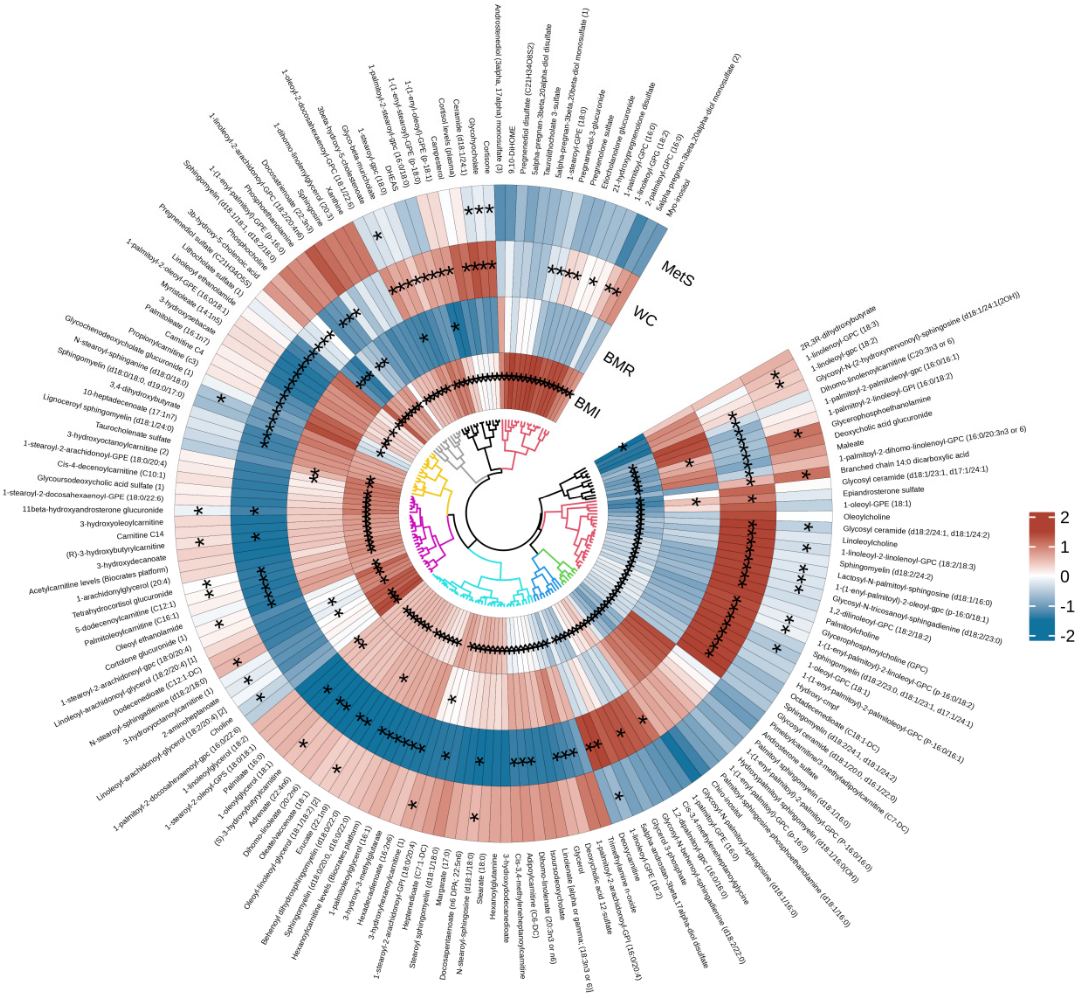

Figure S4. Circular heat map of lipid metabolites and metabolic disease-related phenotypes. * indicates statistical significance ($P_{fdr} < 0.05$).

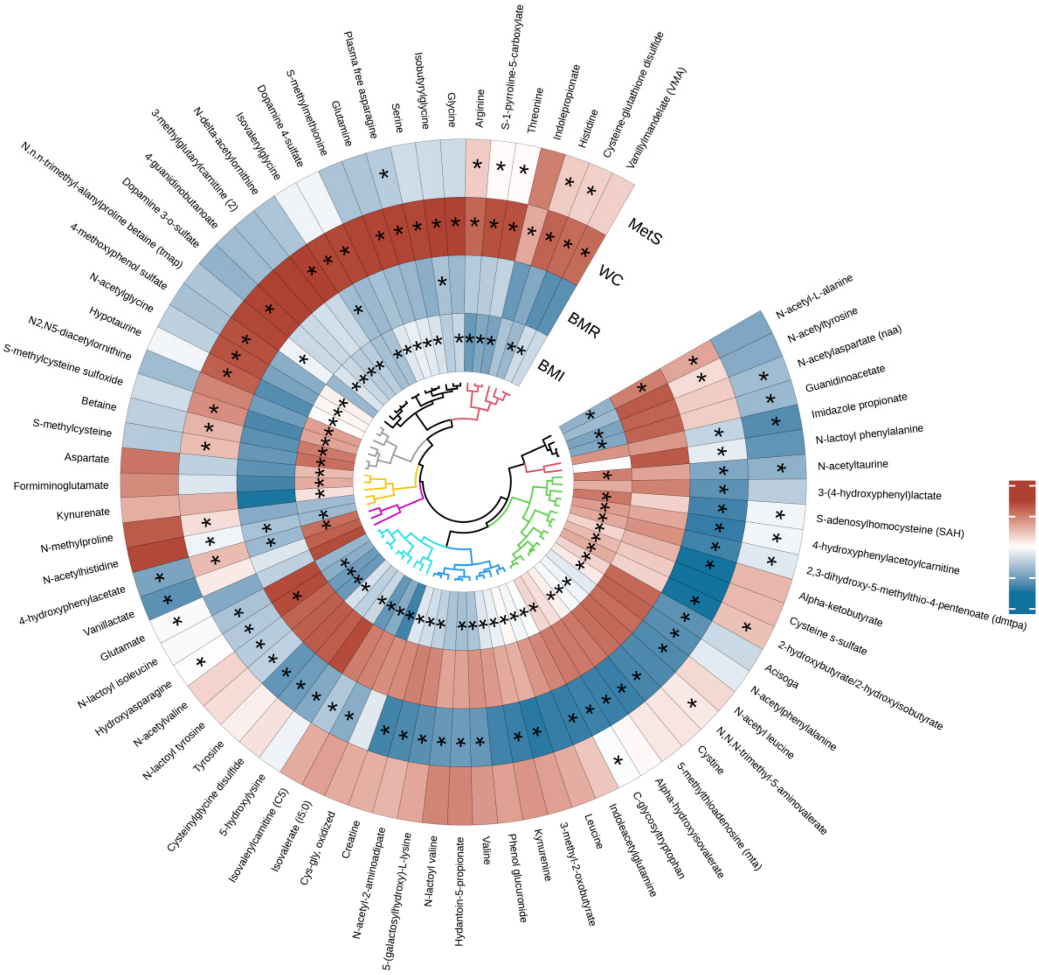

Figure S5. Circular heat map of amino acid metabolites and metabolic disease-related phenotypes. * indicates statistical significance ($P_{fdr} < 0.05$).

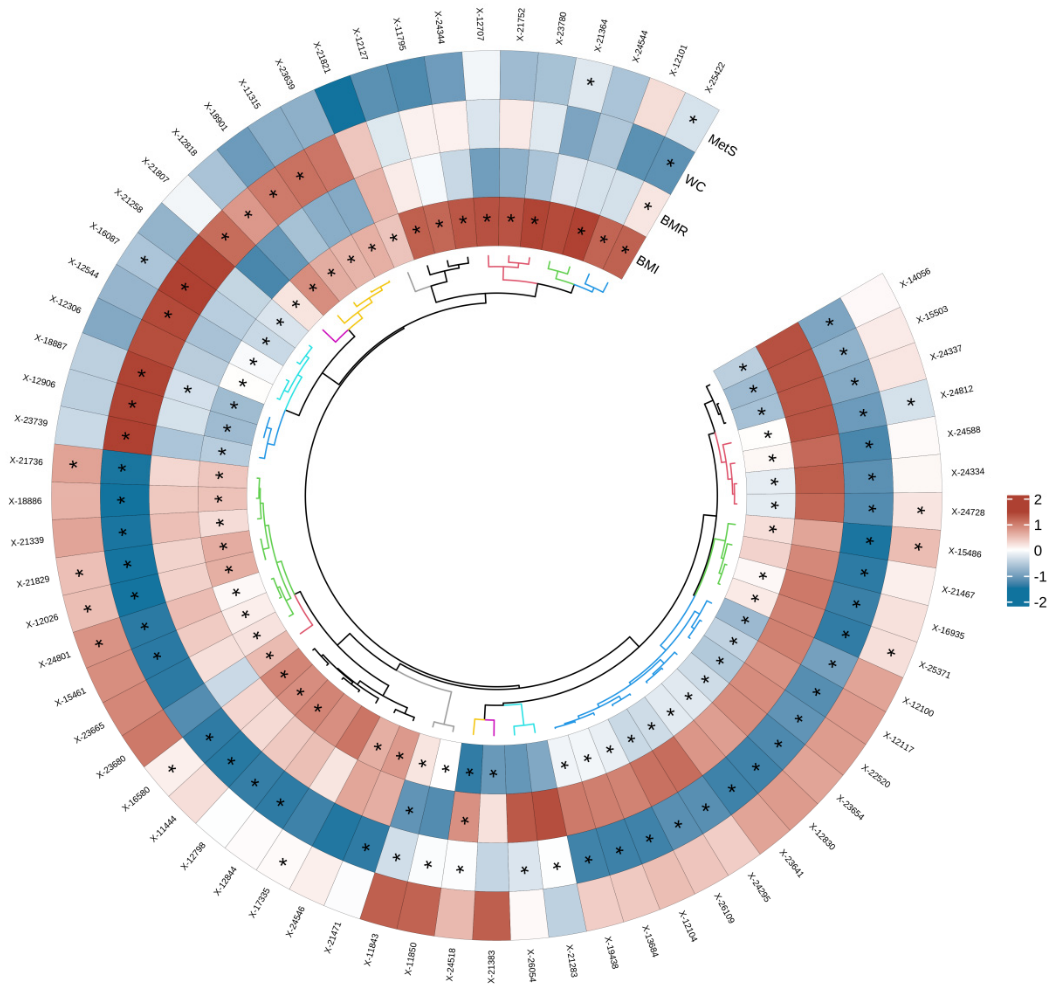

Figure S6. Circular heat map of unknown metabolites and metabolic disease-related phenotypes. * indicates statistical significance ($P_{fdr} < 0.05$).

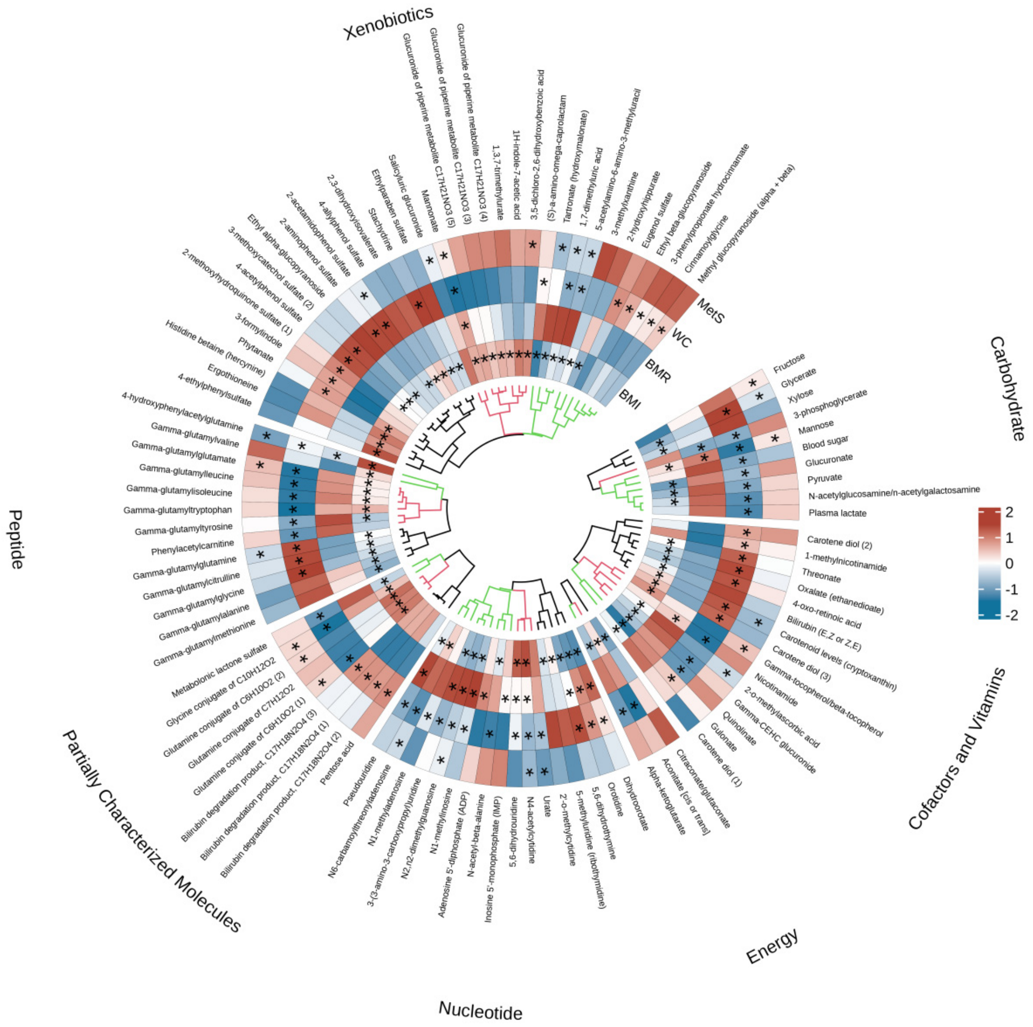

Figure S7. Circular heat map of 6 types of metabolites (xenobiotics, carbohydrate, cofactors and vitamins, peptide, energy, nucleotide and partially characterized molecules) and metabolic disease-related phenotypes.

* indicates statistical significance ($P_{fdr} < 0.05$).

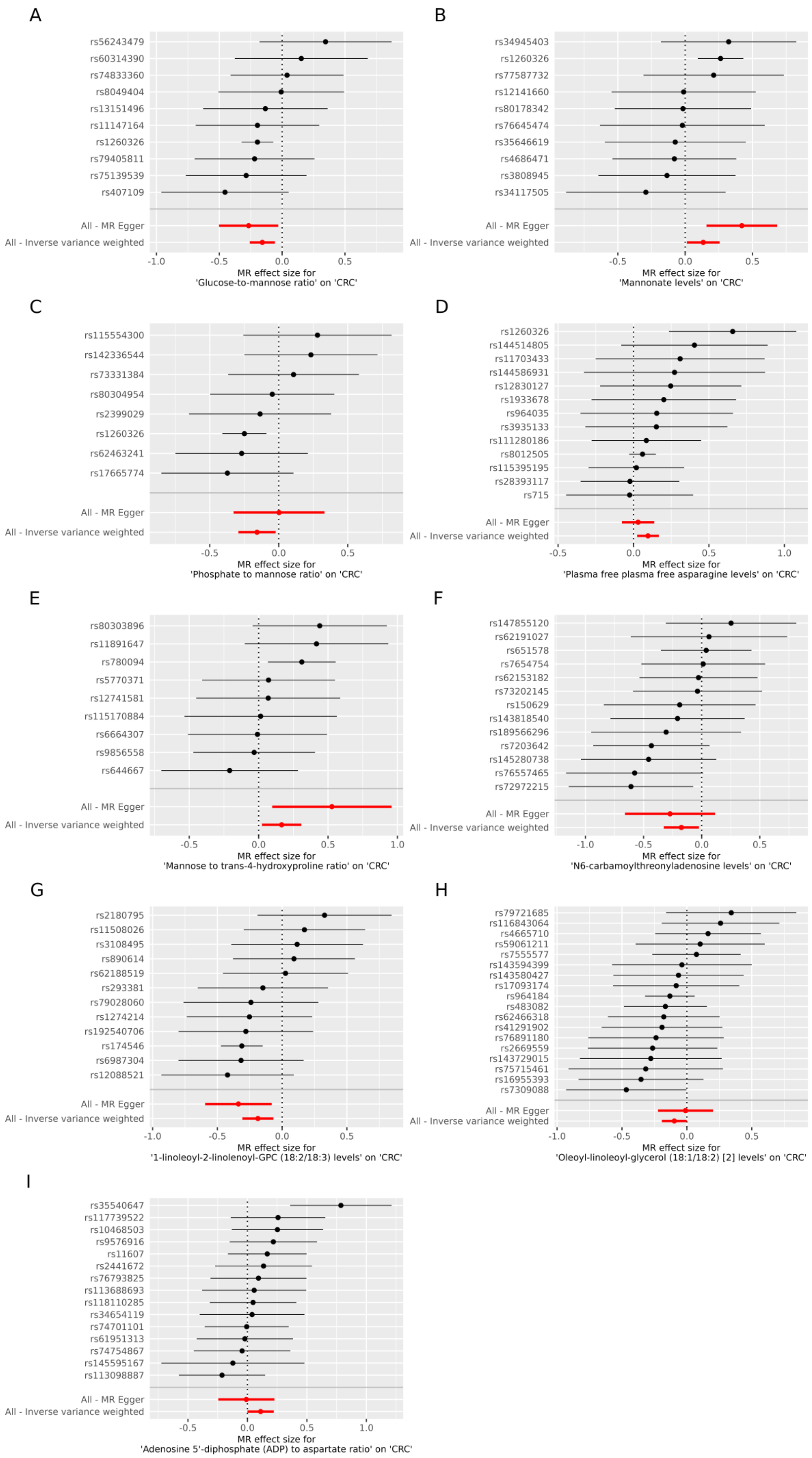

Figure S8. Leave-One-Out plot of effects of intermediate metabolites on colorectal cancer. (A). Effect of

glucose-to-mannose ratio on colorectal cancer. **(B)**. Effect of mannonate levels on colorectal cancer. **(C)**. Effect of phosphate to mannose ratio on colorectal cancer. **(D)**. Effect of Plasma free asparagine levels on colorectal cancer. **(E)**. Effect of mannose to trans-4-hydroxyproline ratio on colorectal cancer. **(F)**. Effect of N6-carbamoylthreonyladenosine levels on colorectal cancer. **(G)**. Effect of 1-linoleoyl-2-linolenoyl-GPC (18:2/18:3) levels on colorectal cancer. **(H)**. Effect of Oleoyl-linoleoyl glycerol (18:1/18:2) levels on colorectal cancer. **(I)**. Effect of Adenosine 5' diphosphate (ADP) to aspartate ratio on colorectal cancer.

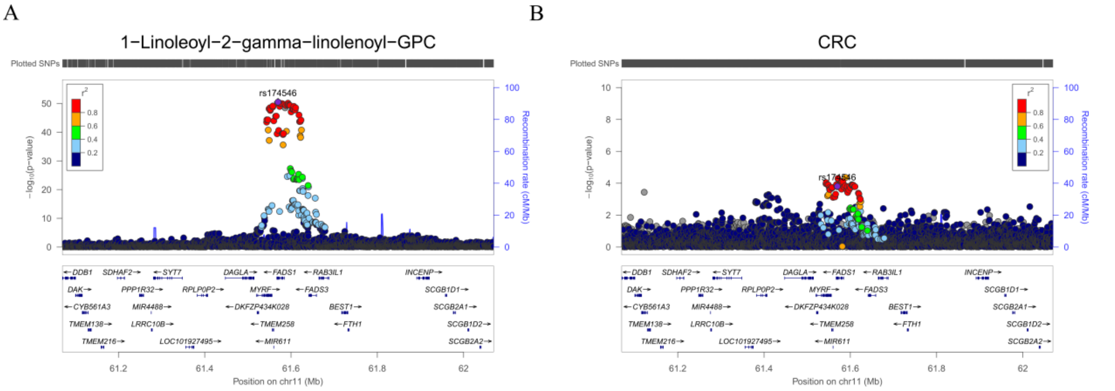

Figure S9. LocusZoom plots of 1-linoleoyl-2-linolenoyl-GPC (18:2/18:3) levels and colorectal cancer. A. LocusZoom plot for 1-linoleoyl-2-linolenoyl-GPC (18:2/18:3) levels GWAS; B. LocusZoom plot for colorectal cancer GWAS. Y-axis on the left represents $\log_{10}(P\text{-value})$ from the two-tailed Z test. The LocusZoom plots were obtained from the LocusZoom website (http://locuszoom.org/)

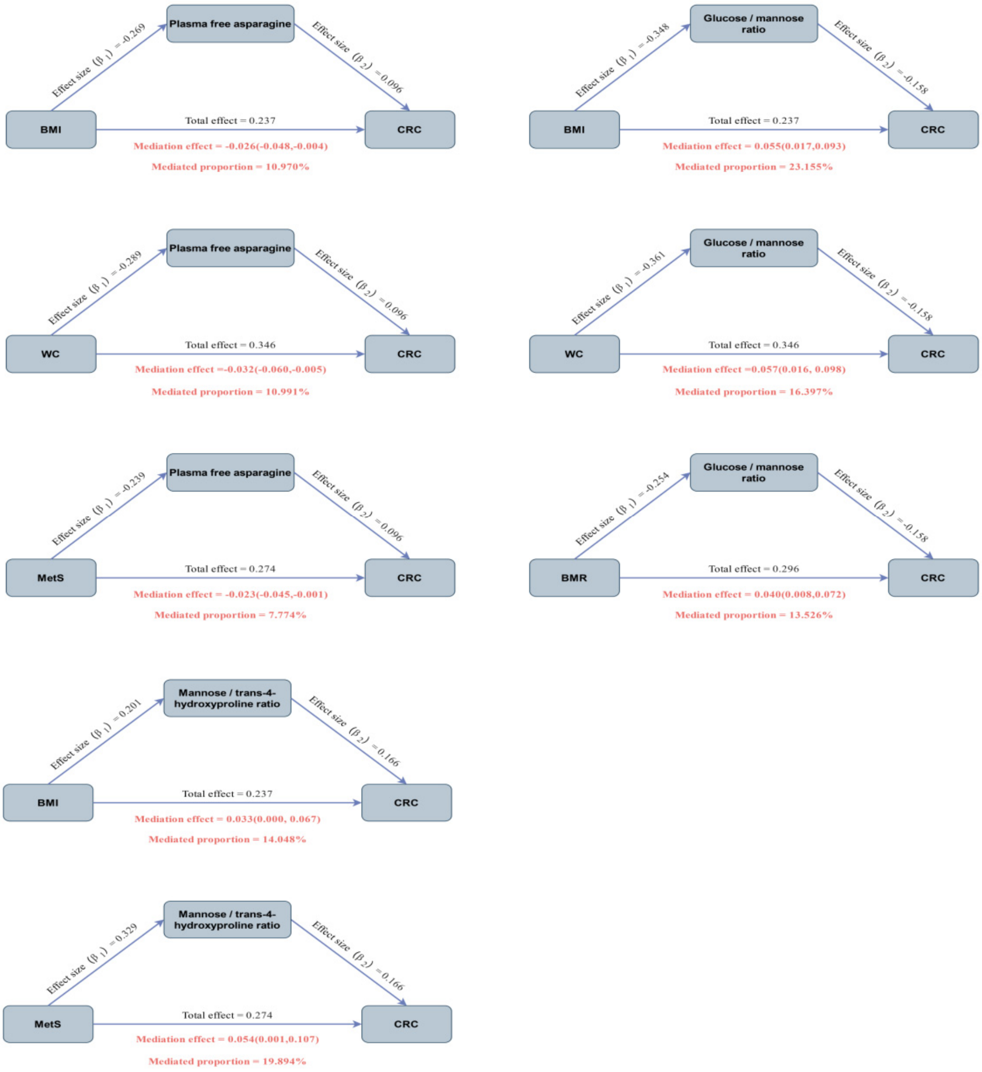

Figure S10. Mediating ratios of plasma free asparagine levels, mannose to trans-4-hydroxyproline ratio and glucose-to-mannose ratio in the causal pathway.

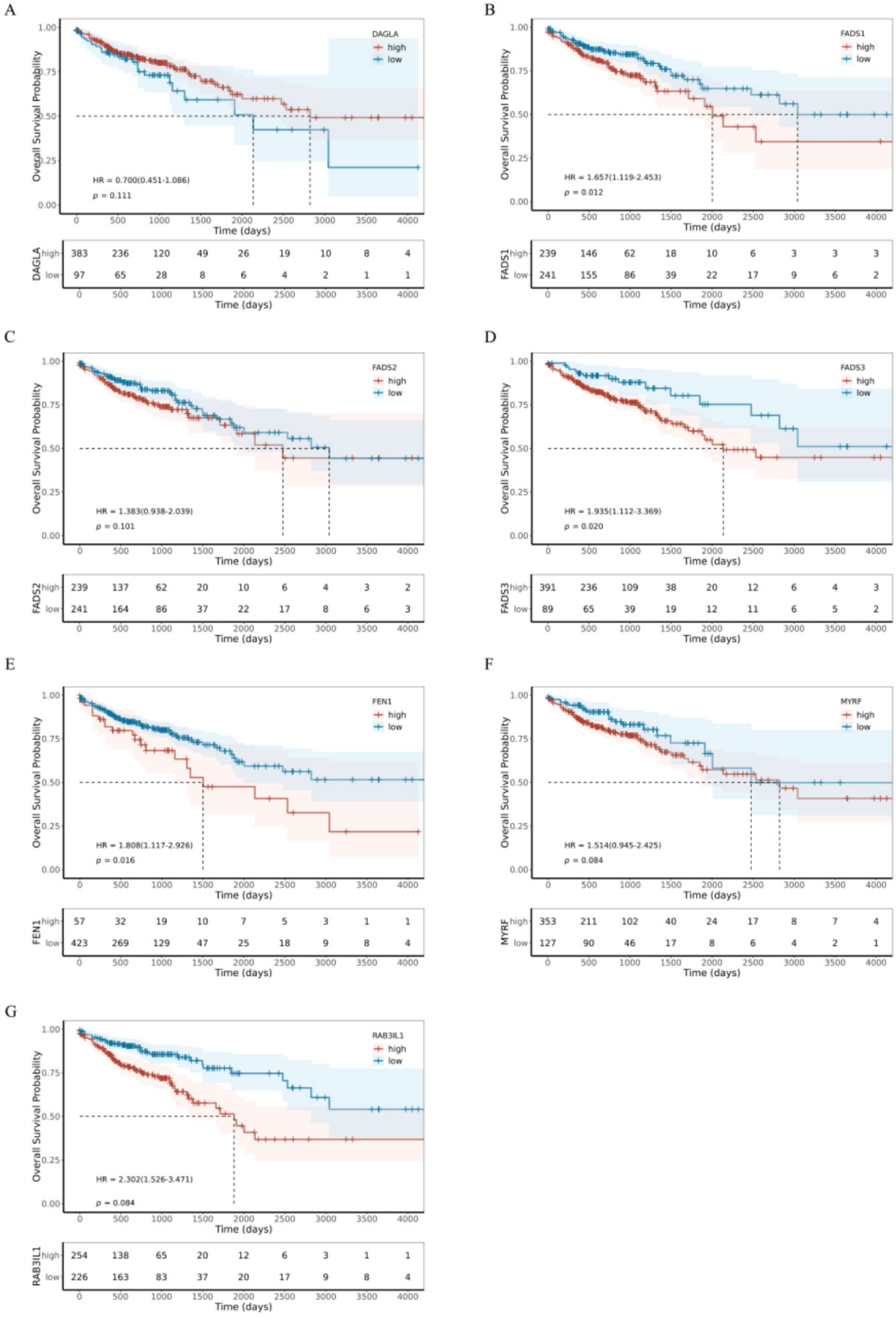

Figure S11. Survival curves for genes near *rs174546*. (A). Survival curve of *DAGLA* **(B)**. Survival curve of *FADS1*. **(C)**. Survival curve of *FADS2*. **(D)**. Survival curve of *FADS3*. **(E)**.Survival curve of *FEN1*. **(F)**. Survival

curve of *MYRF*. **(G)**. Survival curve of *RAB3IL1*. HR and confidence intervals from univariate Cox proportional hazards regression, *P* values from log-rank test

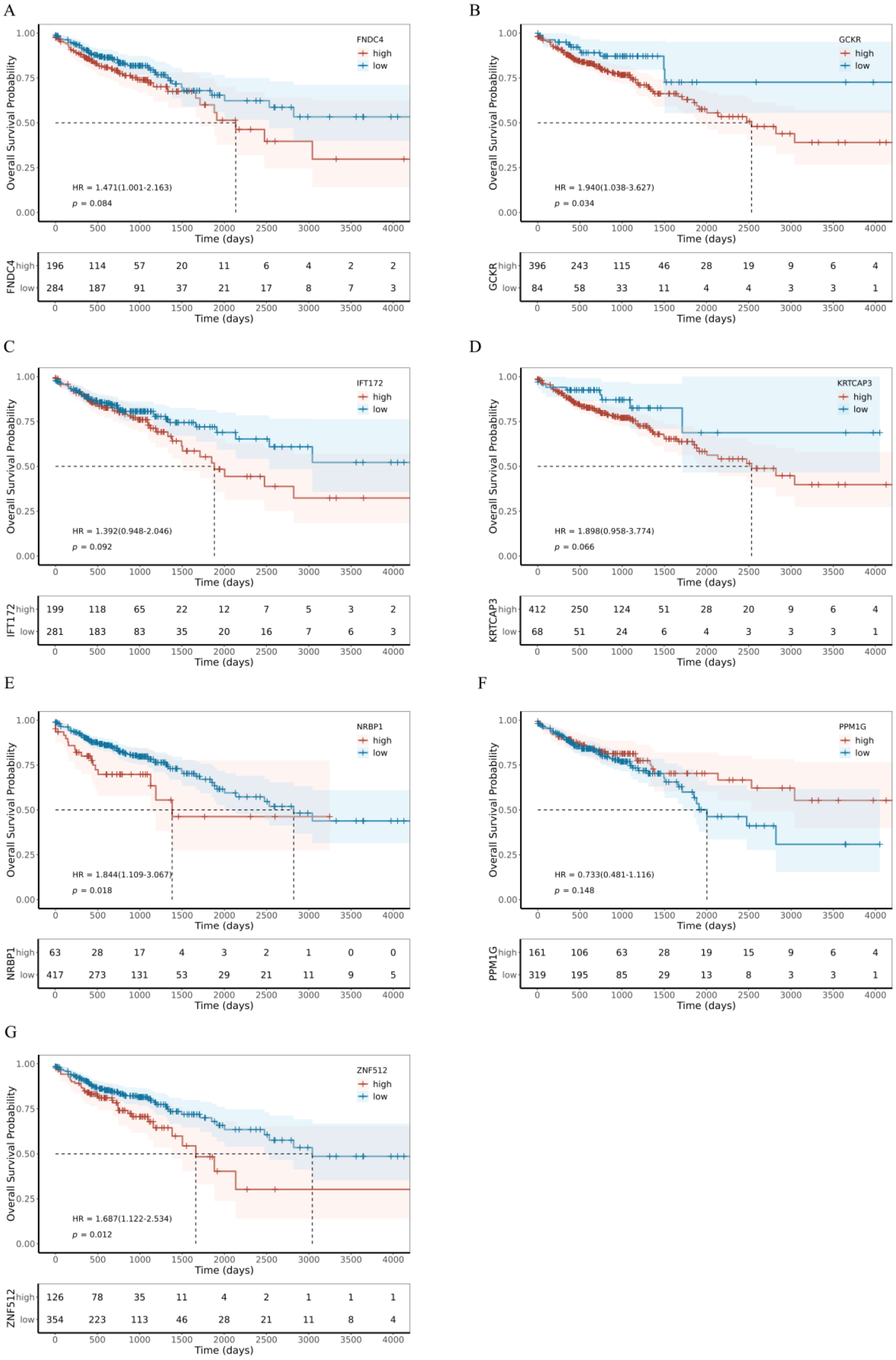

Figure S12. Survival curves for genes near *rs1260326*. (A). Survival curve of *FNDC4* **(B)**. Survival curve of *GCKR*. **(C)**. Survival curve of *IFT172*. **(D)**. Survival curve of *KRTCAP3*. **(E)**.Survival curve of *NRBP1*. **(F)**. Survival curve of *PPM1G*. **(G)**. Survival curve of *ZNF512*. HR and confidence intervals from univariate Cox

proportional hazards regression, *P* values from log-rank test

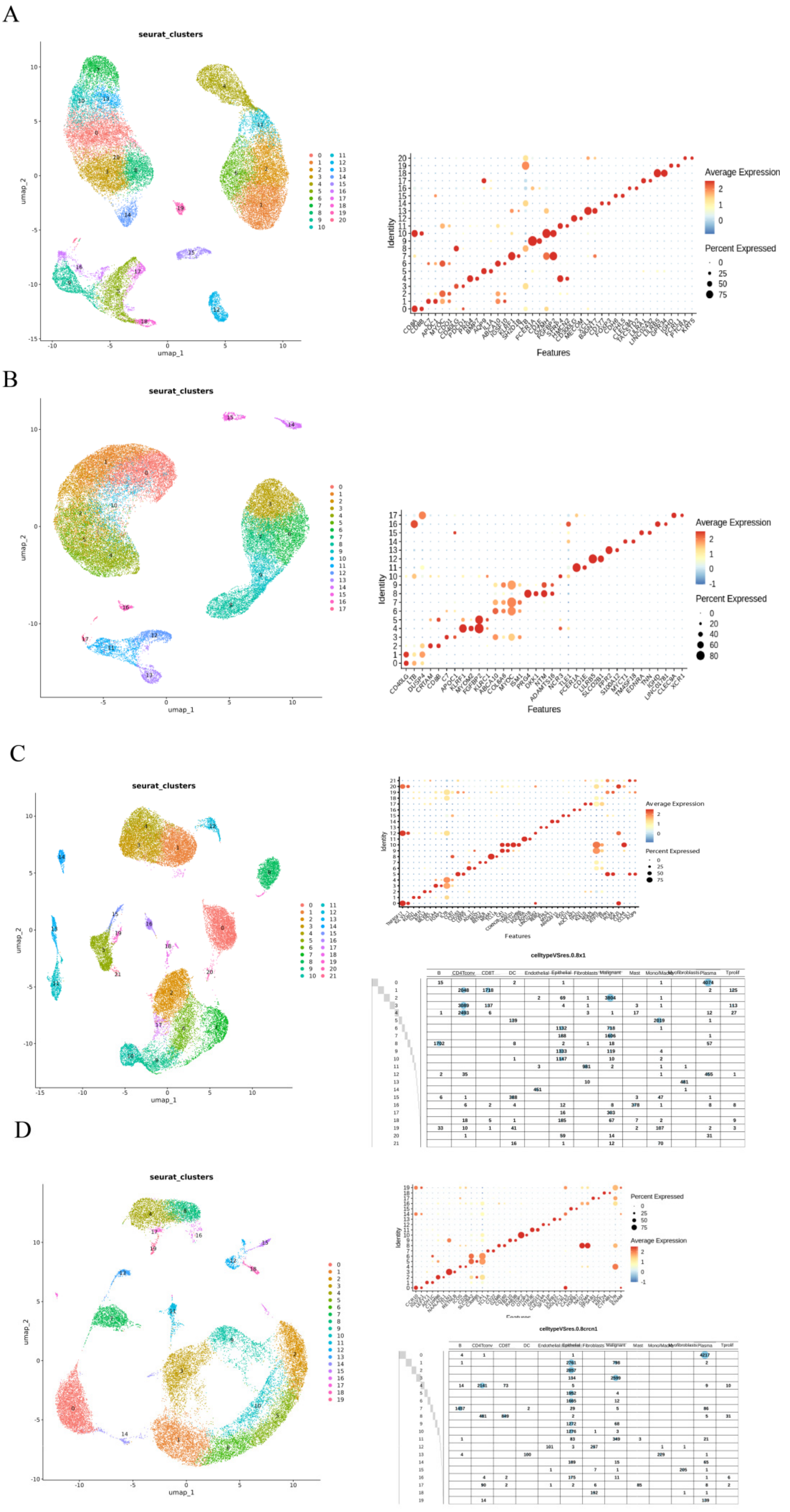

celltypeVSres.0.8x1

	B	CD4Tconv	CD8T	DC	Endothelial	Epithelial	Fibroblasts	Malignant	Mast	Mono/Mac	Myofibroblasts	Plasma	Tprolif
0	15			2		1				1		4074	
1		2048	1718									2	125
2					2	69	1	3804		1			
3		3089	137			4	1		3	1			113
4	1	2493	6				3	1	17			12	27
5				139						2019		1	
6						1132		718		1			
7						188		1606				1	
8	1702			8		2	1	18				57	
9						1333		119		4			
10				1		1147		10		2			
11					3		981	2		1	1		
12	2	35								1		455	1
13							10				481		
14					451						1		
15	6	1		388					3	47		1	
16		6	2	4		12		8	378	1		8	8
17						16		303					
18		18	5	1		185		67	7	2			9
19	33	10	1	41					2	107		2	3
20		1				59		14				31	
21				16		1		12		70			

celltypeVSres.0.8crcn1

	B	CD4Tconv	CD8T	DC	Endothelial	Epithelial	Fibroblasts	Malignant	Mast	Mono/Mac	Myofibroblasts	Plasma	Tprolif
0	4	1				1						4217	
1	1					2761		798				2	
2						2857							
3						134		2599					
4	14	2141	73			5						9	10
5						1952		4					
6						1665		12					
7	1457			2		29		5				86	
8		481	849			2						5	31
9						1272		68					
10						1276	1	3					
11	1					83		349	3			21	
12					101	3	297			1	1		
13	4			100						229		1	
14						189		15				65	
15					1		7	1			205	1	
16		4	2			175		11				1	6
17		90	2		1	2	6		85			8	2
18							192				1	1	
19		14										139	

Figure S13. Single-cell transcriptomic profiling of adipose and colorectal tissues. A. UMAP clustering (left) and dot plot of the top 2 expressed genes (right) in single-cell data from adipose tissue of obese individuals (GSE155960); B. UMAP clustering (left) and dot plot of the top 2 expressed genes (right) in adipose tissue of lean individuals (GSE155960); C. UMAP clustering (left), top 2 expressed genes (upper right), and number of differentially expressed genes per cluster (lower right) in CRC tumor tissue (GSE166555); D. Same analyses as in (C) for adjacent normal colorectal tissue;
